# Supplementary material for: Small in size, big on taste: Metabolomics analysis of flavor compounds from Philippine garlic
Source: PLoS One. 2021 May 20;16(5):e0247289. doi: 10.1371/journal.pone.0247289 (PMC8136657; doi:10.1371/journal.pone.0247289)
Supplement: S1 Fig — (PDF) [file pone.0247289.s001.pdf]

Supporting Information

Small in Size, Big on Taste: Metabolomics analysis of flavor compounds from Philippine Garlic.

Molino, RJE, Rellin, KF, Nellas, R, Junio, H

S1: Sampling sites and morphometric measurements

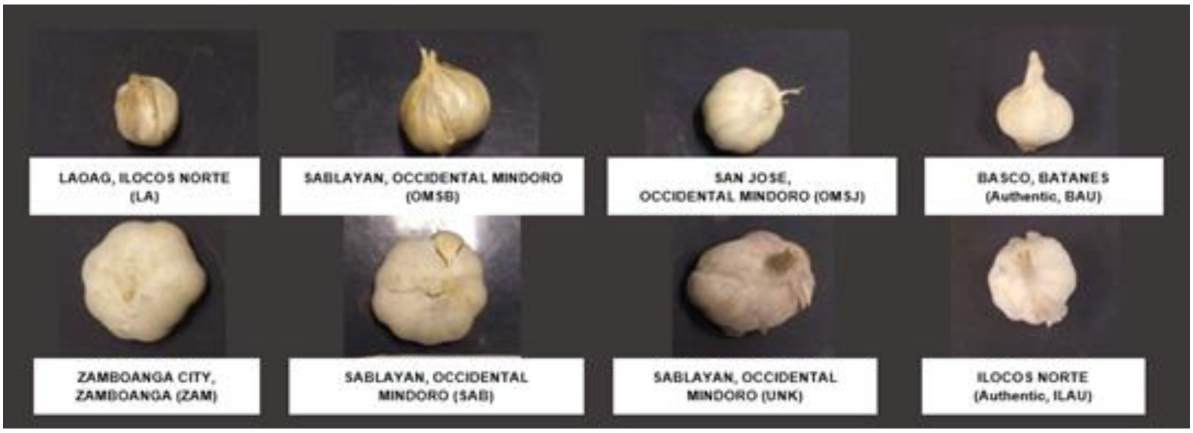

S1 Figure 1. Representative bulbs of local and imported garlic. A major observation is the smaller bulb size and more cloves per bulb in native garlic cultivars.
