# Supplementary material for: Small in size, big on taste: Metabolomics analysis of flavor compounds from Philippine garlic
Source: PLoS One. 2021 May 20;16(5):e0247289. doi: 10.1371/journal.pone.0247289 (PMC8136657; doi:10.1371/journal.pone.0247289)
Supplement: S2 Fig — (PDF) [file pone.0247289.s002.pdf]

S2. Representative metabolite profiles

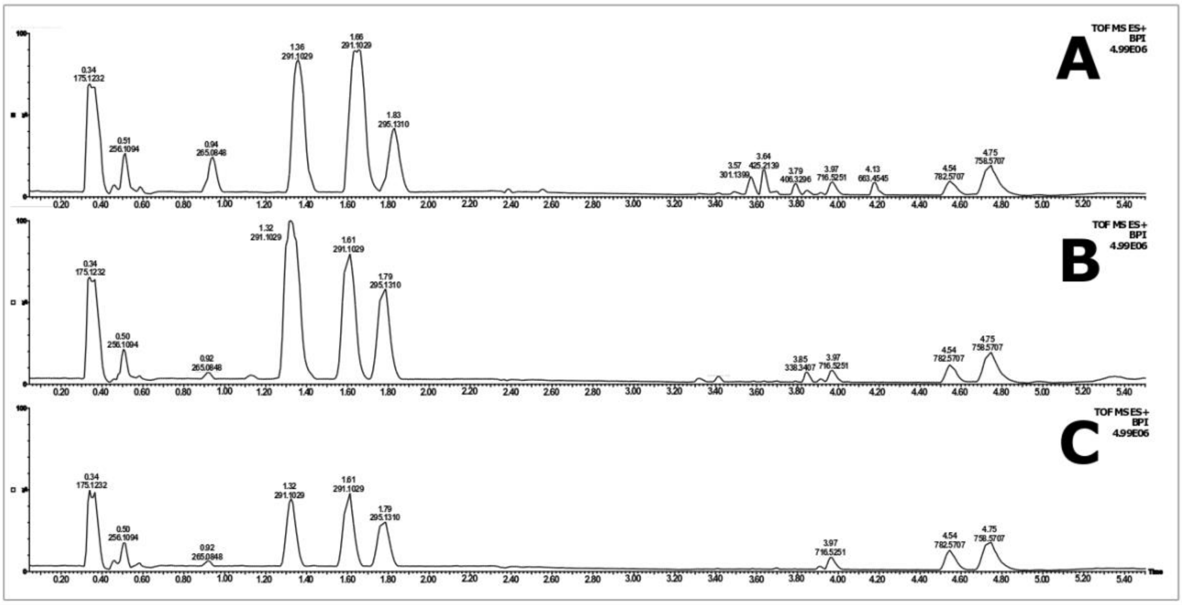

S2 Figure 1: Representative base peak chromatograms of aqueous extracts of authenticated Batanes (A) and Ilocos garlic (B) as well as a market sample from Quezon City (C). Higher levels of dipeptides were extracted compared to the ethanolic extract (Supplementary Info D) although same compounds were annotated on both extracts.

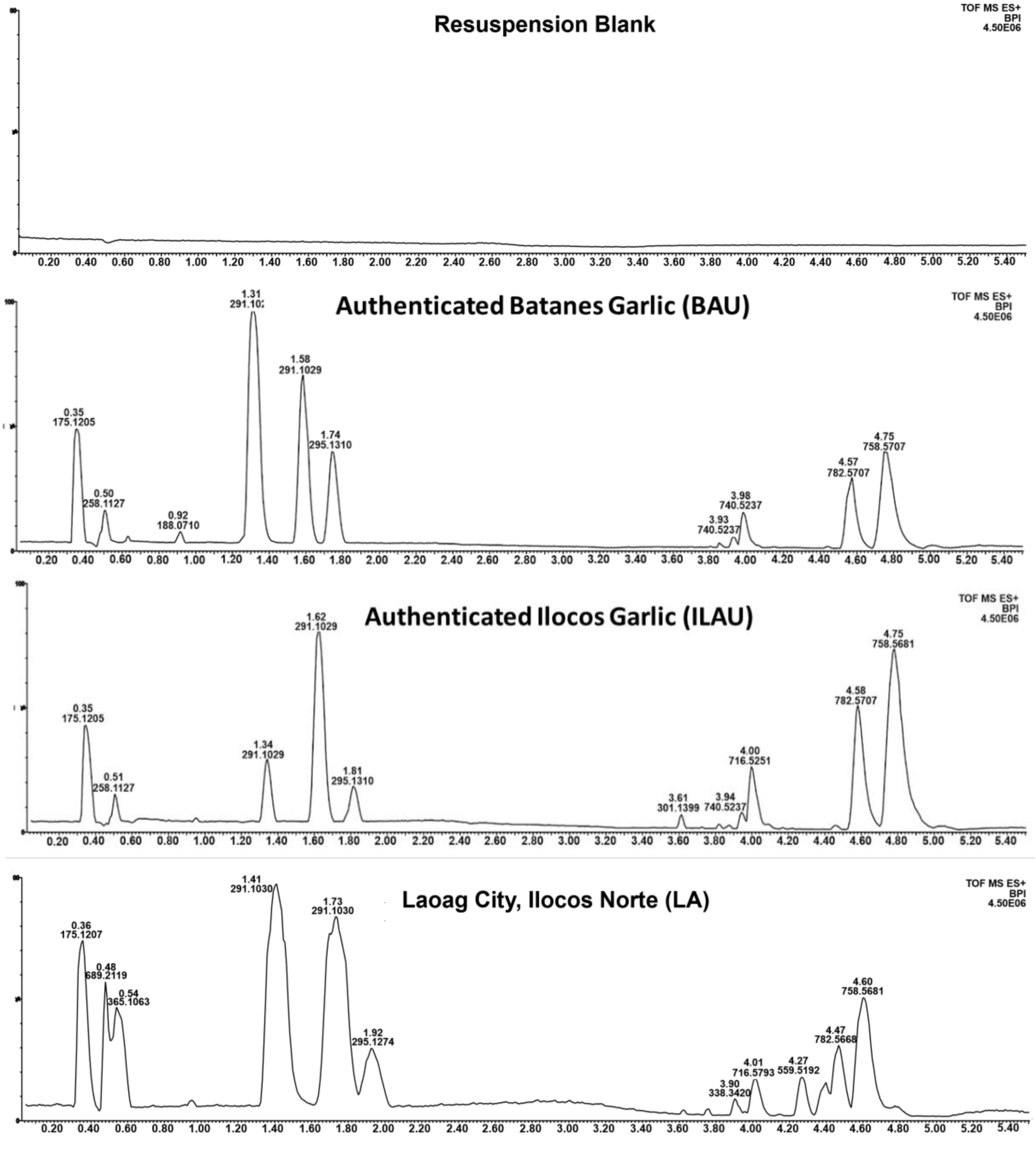

S2 Figure 2. LC-MS profiles of authenticated samples and representative market. Base peak ion chromatogram of resuspension blank, authenticated Batanes (BAU), authenticated Ilocos (ILAU), and Laoag Market (LA) garlic samples.

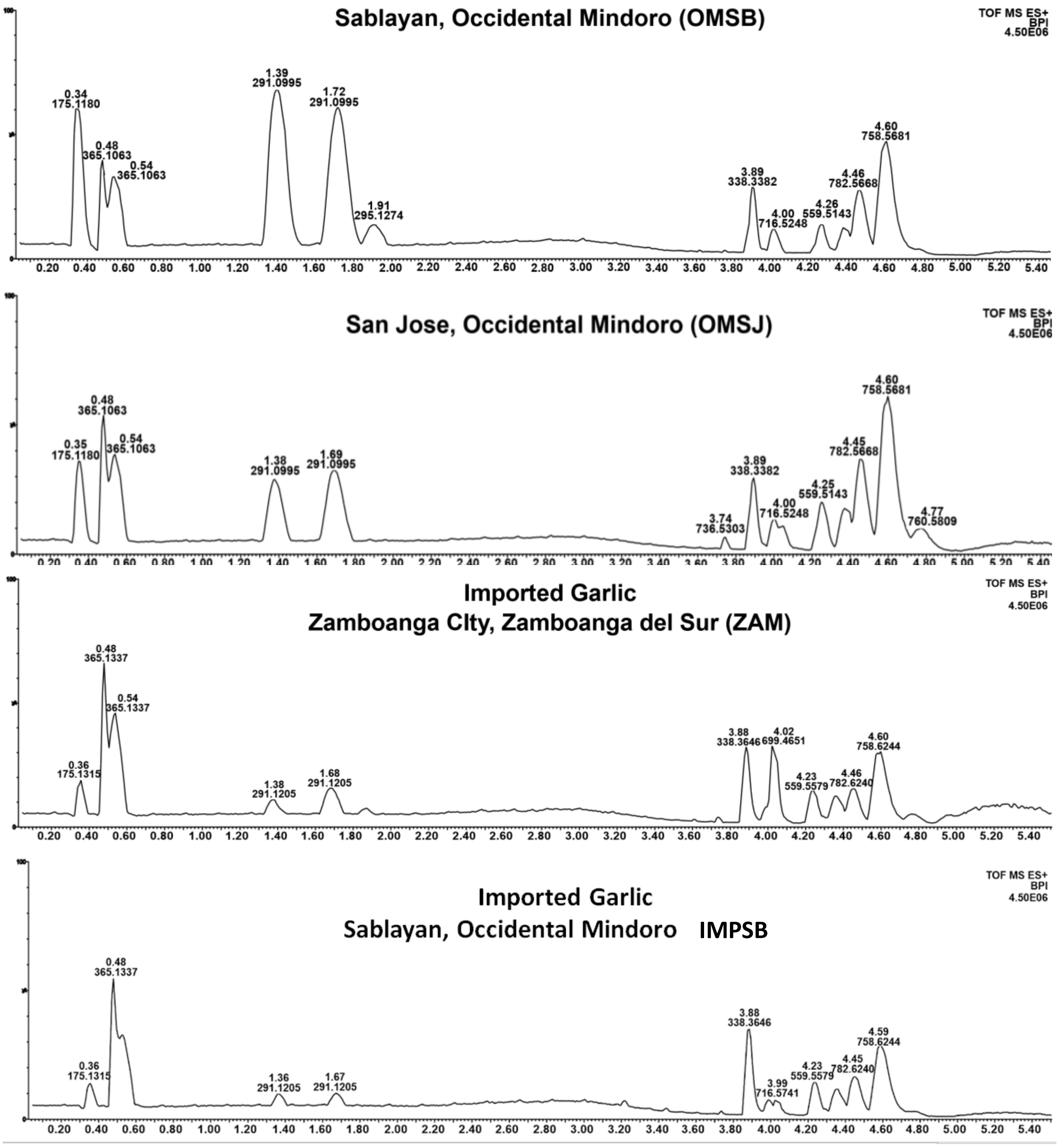

S2 Figure 3. LC-MS profiles of local and imported market samples. Base peak ion chromatogram of native garlic from Sablayan, Occ. Mindoro (OMSB) and San Jose, Occ. Mindoro (OMSJ) as well as imported garlic from Zamboanga City, Zamboanga del Sur (ZAM), and Sablayan, Occidental Mindoro (IMPSB).

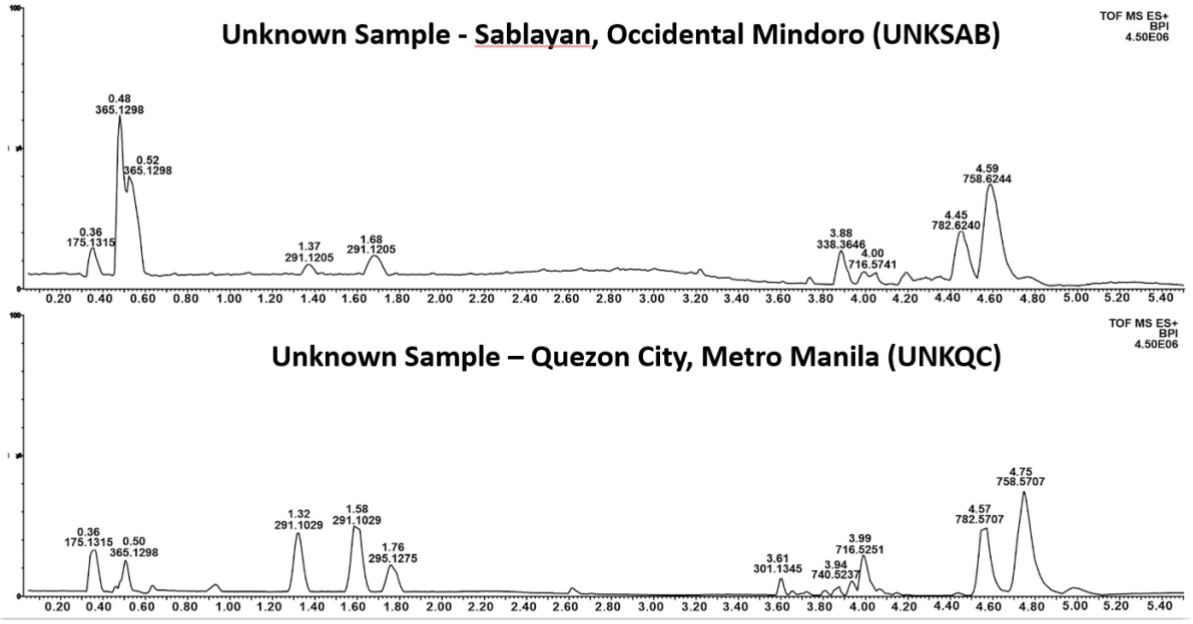

S2 Figure 4. LC-MS profiles of unknown samples. Base peak ion chromatogram of unknown garlic samples collected in Sablayan, Occidental Mindoro (UNKSAB) and Quezon City, Metro Manila (UNKQC)
