# Supplementary material for: Small in size, big on taste: Metabolomics analysis of flavor compounds from Philippine garlic
Source: PLoS One. 2021 May 20;16(5):e0247289. doi: 10.1371/journal.pone.0247289 (PMC8136657; doi:10.1371/journal.pone.0247289)
Supplement: S3 Fig — (PDF) [file pone.0247289.s003.pdf]

S3. Molecular networking and structural analysis

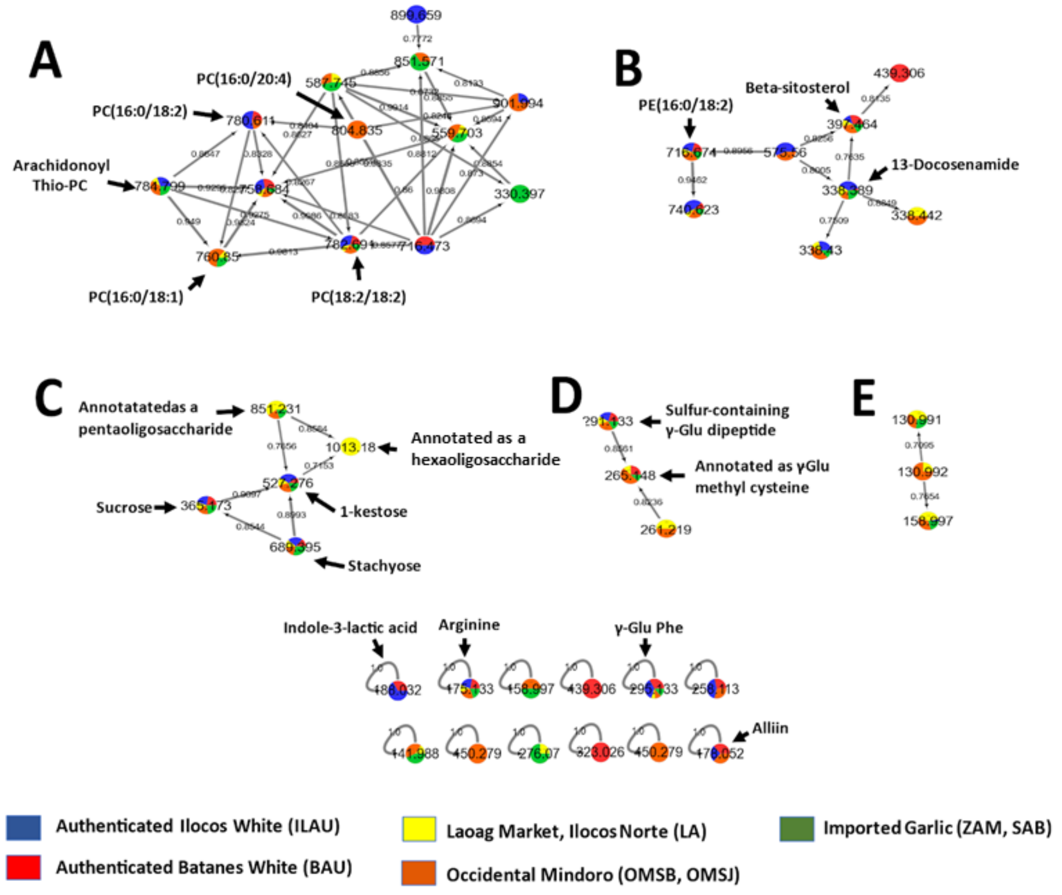

S3 Figure 1. Molecular network obtained from DDA analysis of local and imported garlic cultivars. Metabolites are represented as nodes, which are connected if the cosine score (edge label and thickness) between the consensus mass spectra exceeds a value of 0.70. The pie graphs, unique for each node, represent the relative abundance of the precursor ions among the different garlic samples. Network (A) contains putatively identified phosphocholine-type lipids; (B) has phosphoethanolamine-type lipids and fatty acids; (C) a network of oligosaccharides; (D) consists of three annotated γ-Glu dipeptides while (E) has mass spectra with no library hits in GNPS.

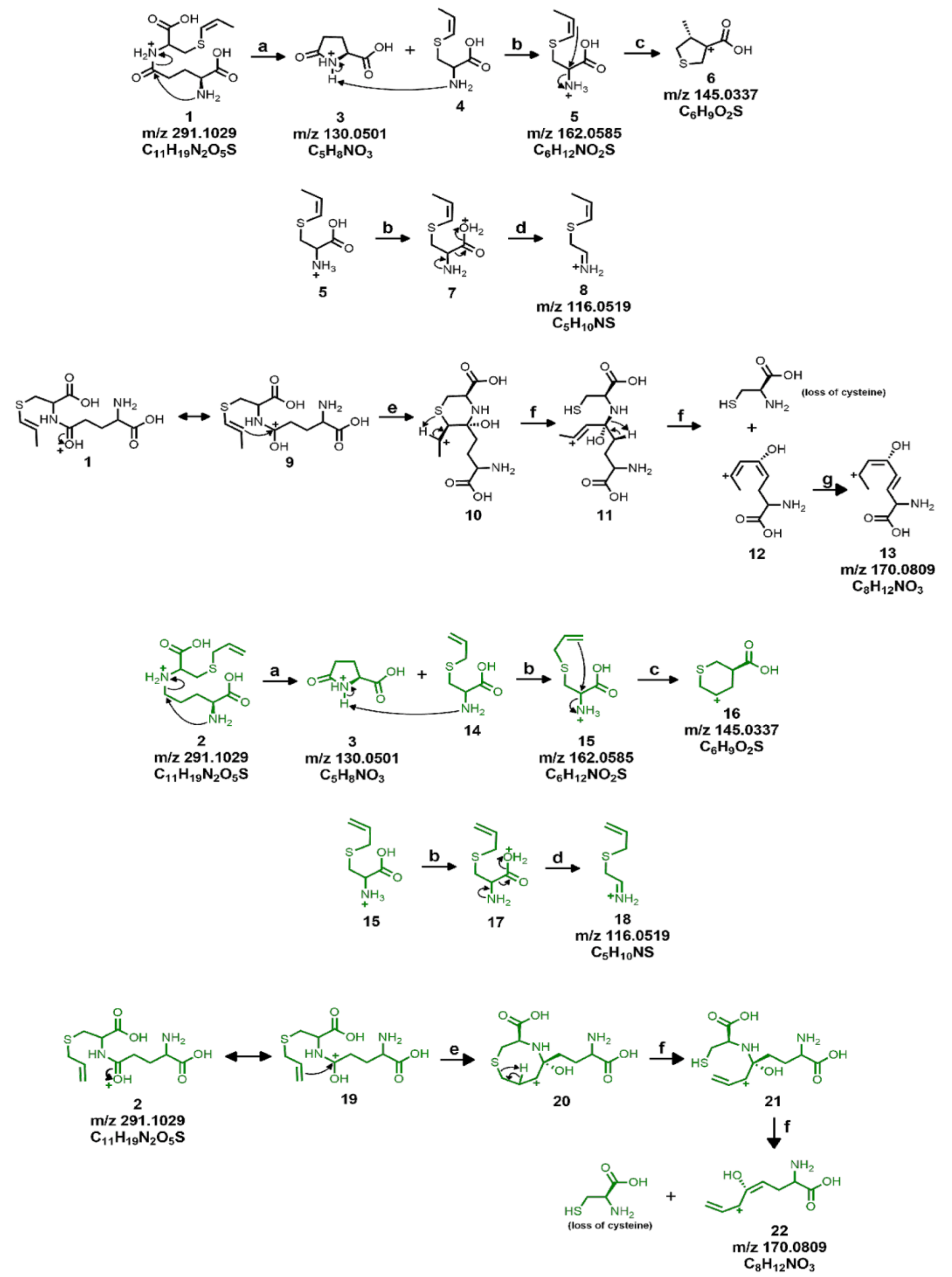

S3 Figure 2. Proposed fragmentation for select product ions of γ-glutamyl propenyl cysteine and γ-glutamyl allyl cysteine. The fragmentation mechanisms for γ-Glu propenyl cysteine are shown in black, while that of γ-Glu allyl cysteine are also shown (green). For the reactions, (a) amide bond formation (b) loss of NH3 through inductive cleavage (c) H+ transfer (d) loss of H2O through inductive cleavage (e) loss of CO (f) Double bond attack to a carbocation, followed by cyclization, and (g) bond cleavage through remote H-rearrangements.

A proposed mechanism to account for product ions of the isomeric dipeptides is shown in **S1C Fig 3**. Intense product ions from both isomers are m/z 162.08, 145.06, and 130.04, initiated from the cleavage of the amide bond (reaction **a**). A concerted mechanism, meanwhile, has been described for the formation of immonium ions from protonated amino acids (Palzs & Suhal, 2005). Product ion with m/z 116.05 is observed in γ-Glu propenyl-Cys precursor ion (Figure 5B; Figure 6, structure 8) but not γ-Glu allyl-Cys due to closer proximity of the positive charge to a stabilizing double bond (Mahadevi & Sastry, 2013). In addition, (Nakabayashi et al., 2016) was able to show through FT-ICR analysis that m/z 170.08 was a characteristic product n of γ-Glu propenyl-Cys. This contrast is also observed on the spectra acquired through QTOF in this experiment. The reaction scheme from structures **9-13** suggests how m/z 170.08 came about. This product ion was specifically for protonated γ-Glu propenyl-Cys due to extensive resonance of the diene system that is allylic to a carbocation (structure **13** compared to **22**).

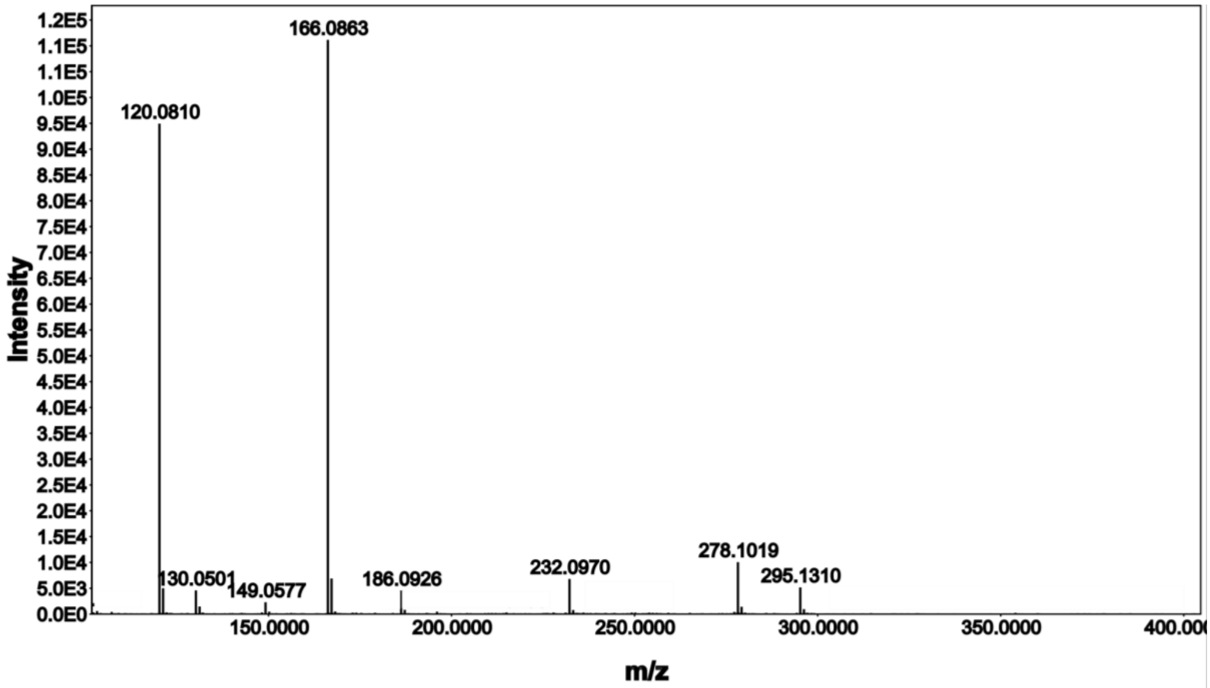

S3 Figure 3. Positive mode MS^2 spectra of m/z 295.1310 putatively identified as γ-Glu Phe by GNPS. The mass spectrum was acquired at 1.74 mins, and 15 eV collision energy was used to obtain the fragment ions. The Highly abundant peak m/z 166.09 was associated with the b_1 ion which includes the phenylalanine moiety. The weakly abundant m/z 130.05 was linked to the y_1 ion expected of γ-Glu dipeptides. The m/z 120.08 ion was related to the immonium ion from phenylalanine.

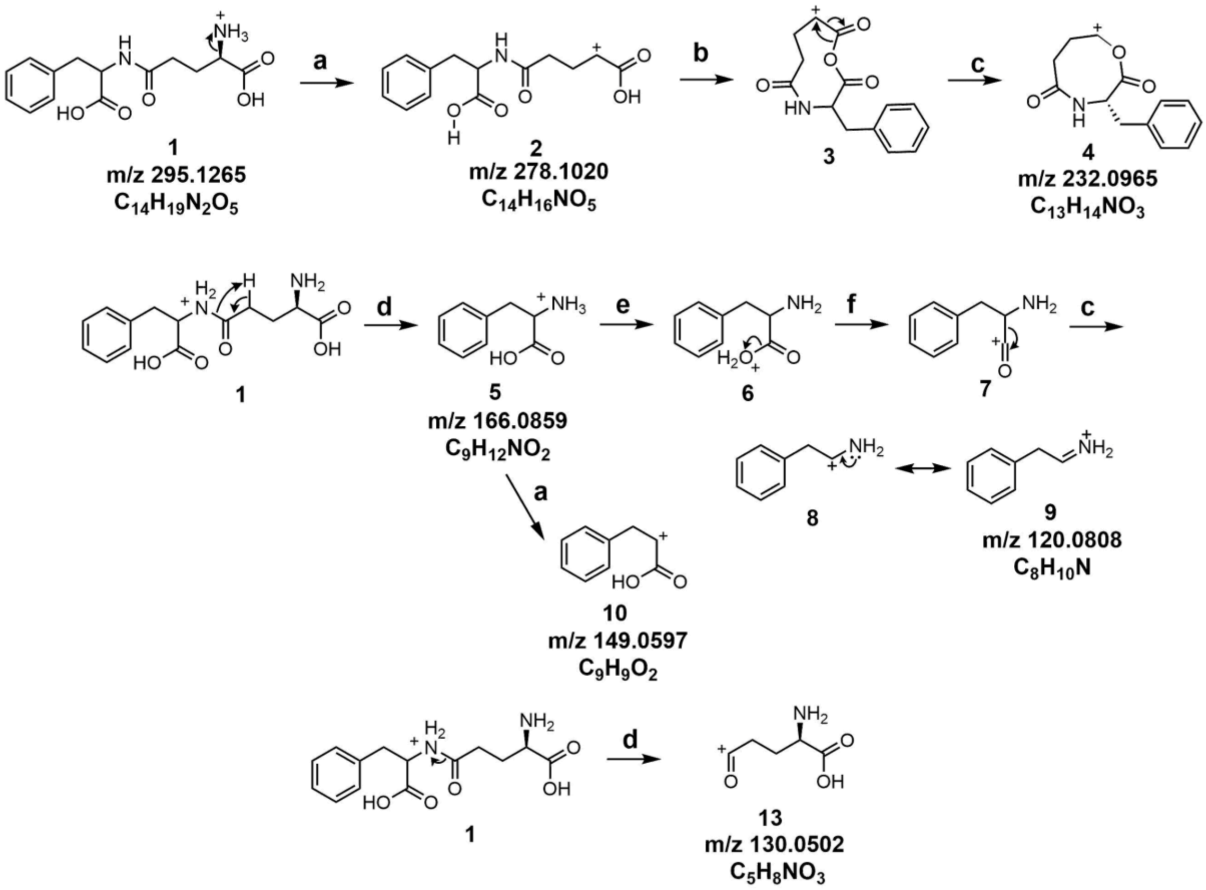

S3 Figure 4. Proposed fragmentation scheme for γ-Glutamyl phenylalanine. The compound was also putatively identified in GNPS. The fragmentation analysis of compounds was still undertaken, in order to provide a reference for the annotation of other γ-Glutamyl dipeptides. For the reactions, (a) corresponds to a loss of NH_3 by inductive cleavage. (b) Cyclization induced by dehydration reaction which occurs via charge retention mechanism (c) loss of CO (d) Formation of y_1- ion (e) mobile H^+ transfer and (f) loss of H_2O via inductive cleavage.

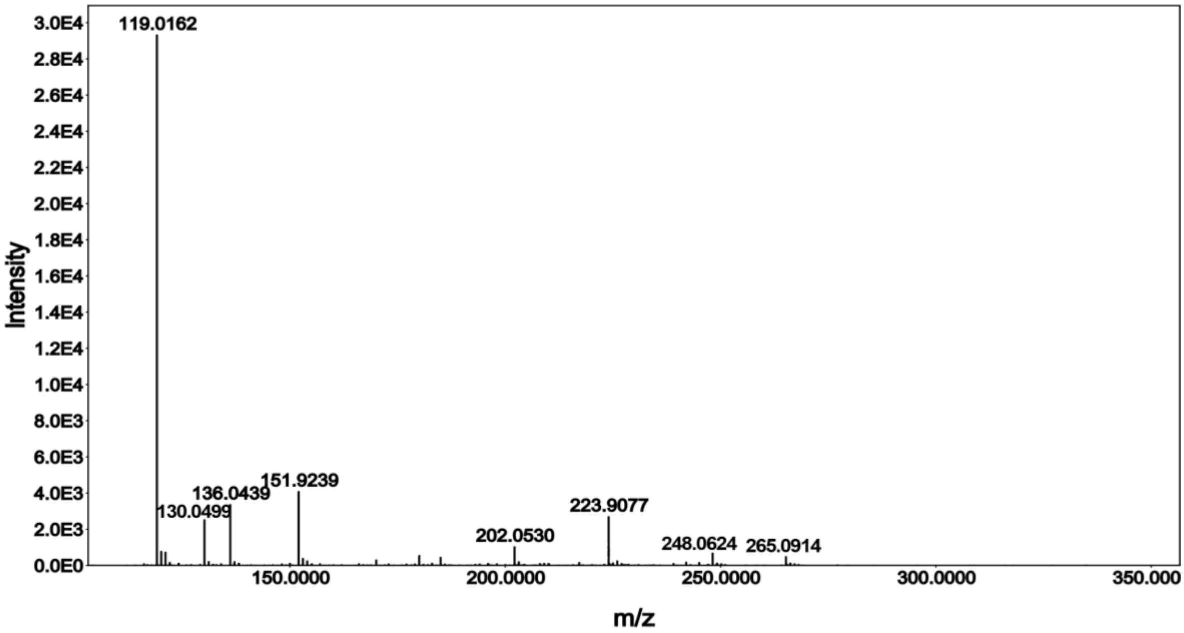

S3 Figure 5. Positive mode MS2 spectra of m/z 265.0894 annotated as γ-Glu Methyl cysteine. The mass spectrum was acquired at 0.93 mins. and 15 eV collision energy was used to obtain the fragment ions. The Highly abundant peak m/z 119.01 was associated with the b_1-NH_3 ion associated with the methyl cysteine moiety. The weakly abundant m/z 130.05 was linked to the y_1 ion expected of γ-Glu dipeptides.

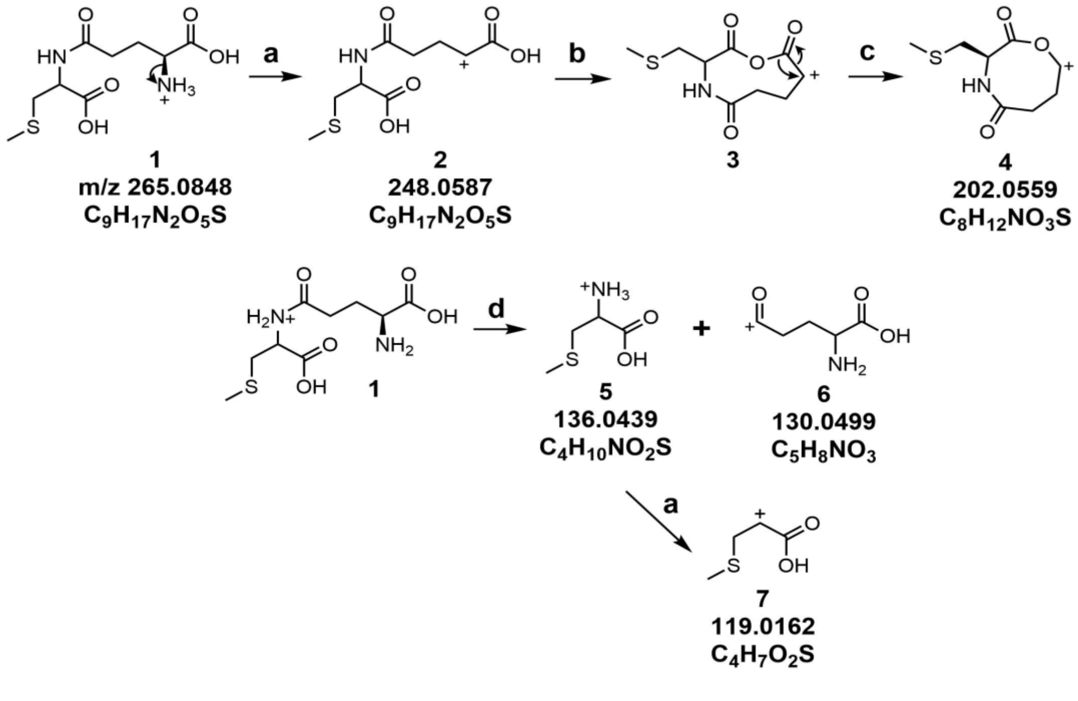

S1C Figure 6. Proposed fragmentation scheme for m/z 265.0894 annotated as γ-Glutamyl methyl cysteine. Fragmentation analysis was critical in the annotation of the m/z 265.09 ion as γ-Glutamyl methyl cysteine. For the reactions, (a) corresponds to a loss of NH_3 by inductive cleavage. (b) Cyclization induced by dehydration reaction which occurs via charge retention mechanism (c) loss of CO, and (d) Formation of y_1-ion.
