# Supplementary material for: Small in size, big on taste: Metabolomics analysis of flavor compounds from Philippine garlic
Source: PLoS One. 2021 May 20;16(5):e0247289. doi: 10.1371/journal.pone.0247289 (PMC8136657; doi:10.1371/journal.pone.0247289)
Supplement: S4 Fig — (PDF) [file pone.0247289.s004.pdf]

S4. Mirror alignment of GNPS-annotated metabolites

Tail-to-tail alignment leading to the putative identification of metabolites from Philippine garlic cultivars. Fragment ions from sample mass spectra is colored in black while that of the reference file GNPS library is in green. The structure, cosine score, and mass error associated with an annotation are also specified. Discrepancies in fragment ion intensities between the sample and reference spectra can be attributed to the differential instruments and settings used to obtain the sample and library data.

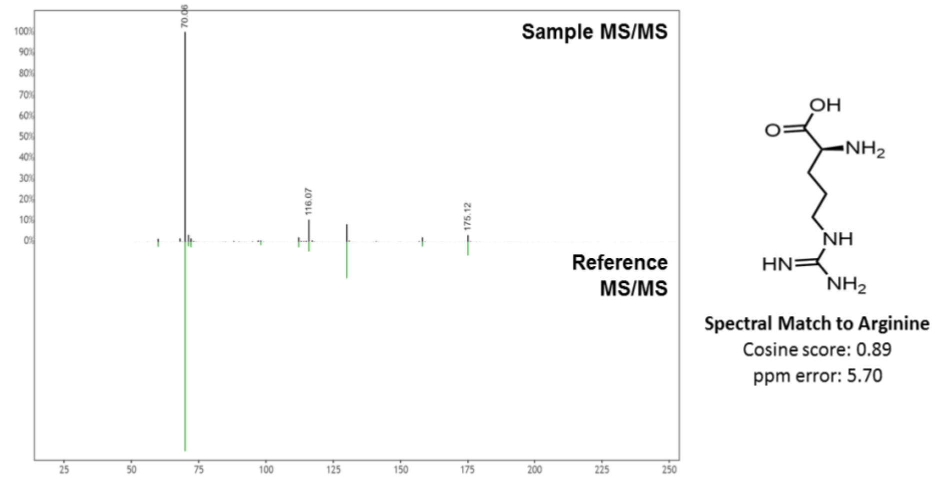

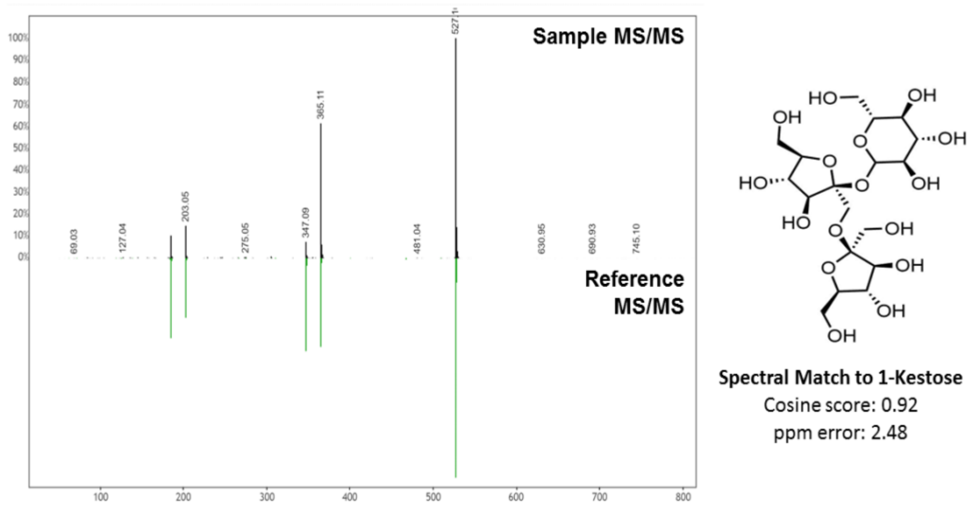

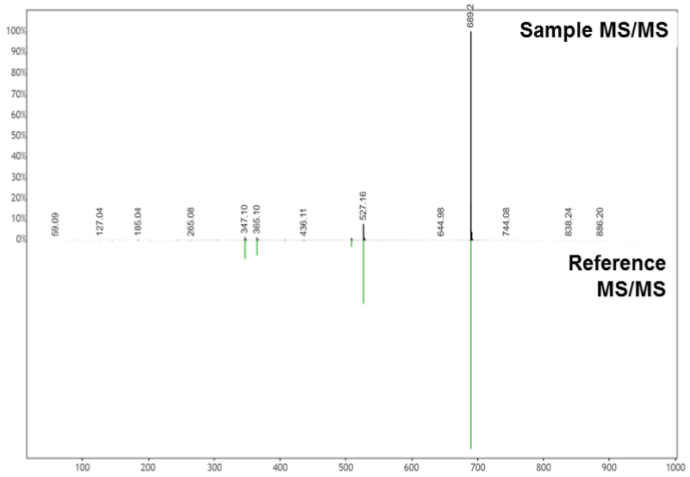

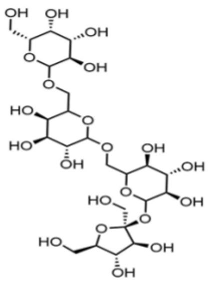

Spectral Match to Stachyose
Cosine score: 0.83
ppm error: 0.39

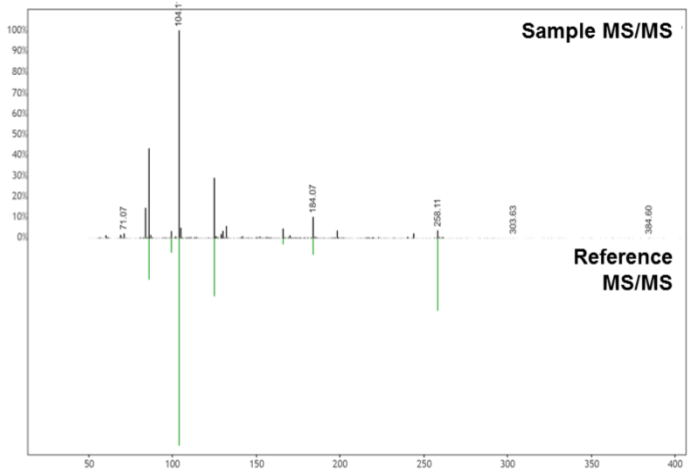

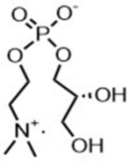

Spectral Match to Sn-glycerol-3-phosphocholine
Cosine score: 0.85
ppm error: 2.52

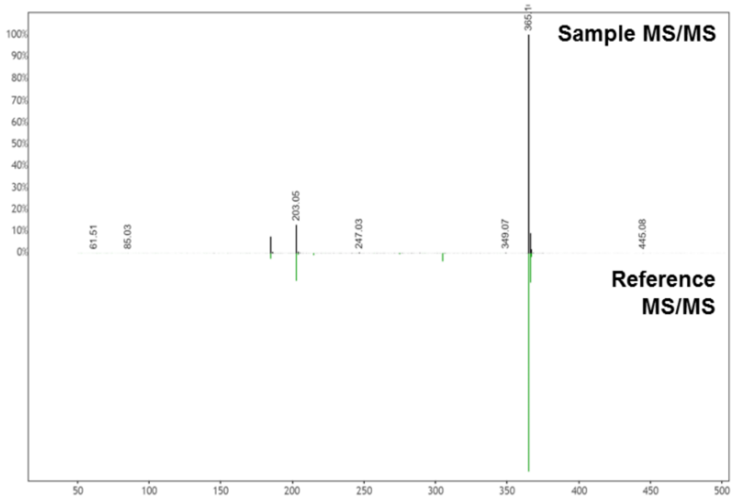

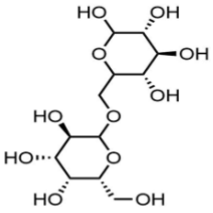

Spectral Match to Melibiose
Cosine score: 0.85
ppm error: 2.52

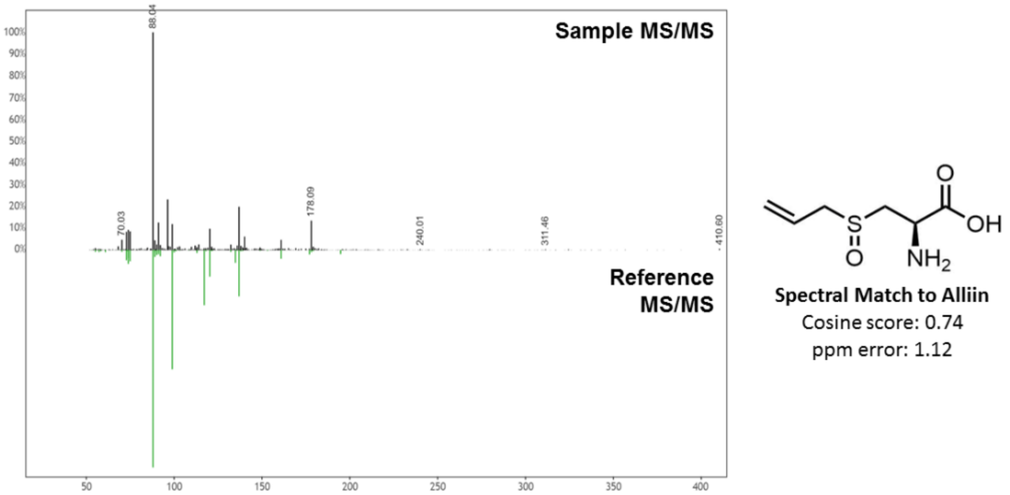
Sample MS/MS
Reference MS/MS
88.04
70.03
178.09
240.01
311.46
410.60
NH2
OH
S
O
Spectral Match to Alliin
Cosine score: 0.74
ppm error: 1.12

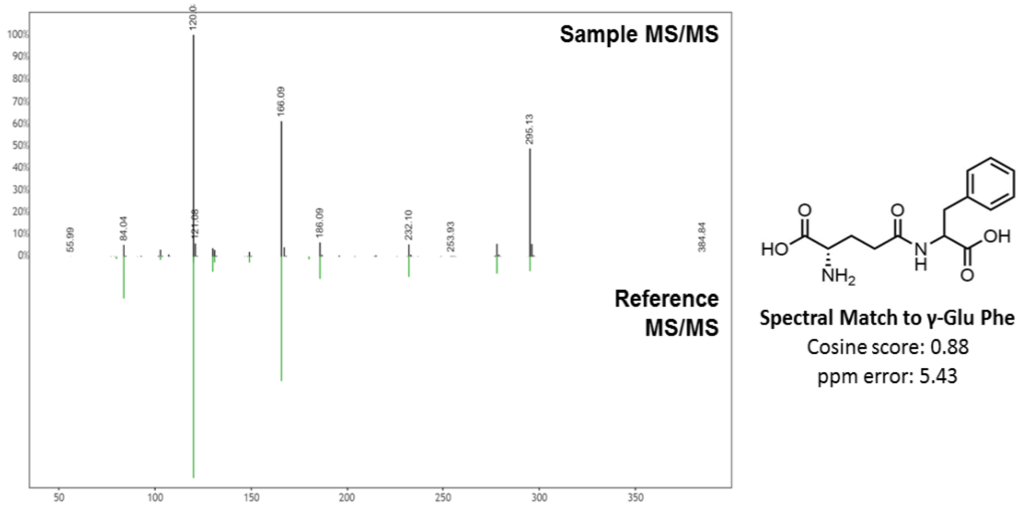
Sample MS/MS
Reference MS/MS
120.0
166.09
295.13
55.99
84.04
121.08
186.09
232.10
253.93
384.84
HO
OH
NH2
N
H
Spectral Match to γ-Glu Phe
Cosine score: 0.88
ppm error: 5.43

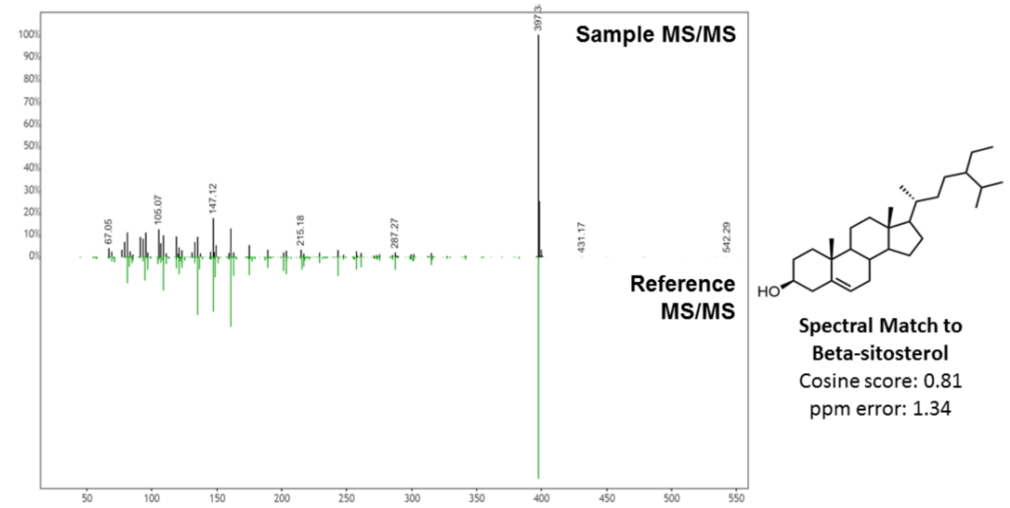
Sample MS/MS
Reference MS/MS
397.3
67.05
105.07
147.12
215.18
287.27
431.17
542.29
HO
Spectral Match to Beta-sitosterol
Cosine score: 0.81
ppm error: 1.34

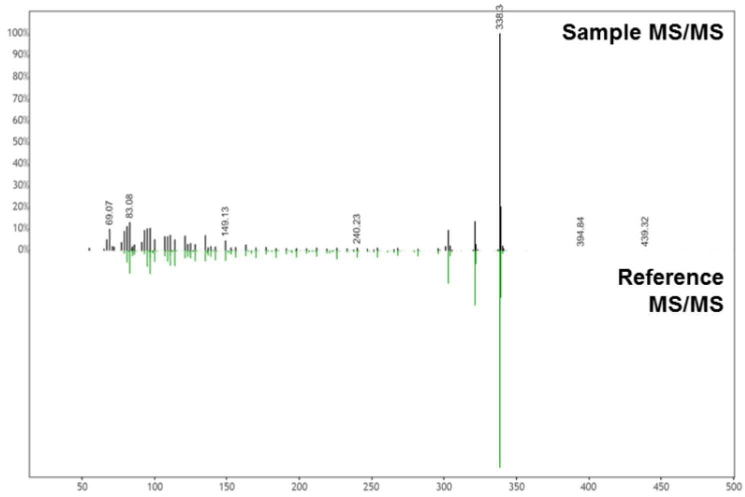

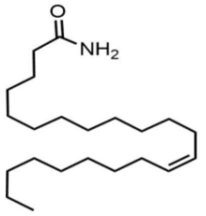

Spectral Match to 13-Docosenamide
Cosine score: 0.86
ppm error: 0.80

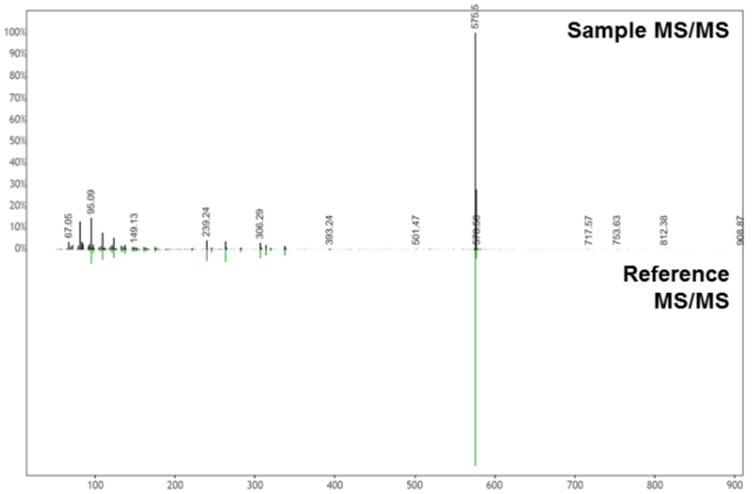

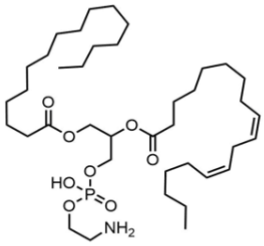

Spectral Match to PE(16:0/18:2)
Cosine score: 0.94
ppm error: 4.19

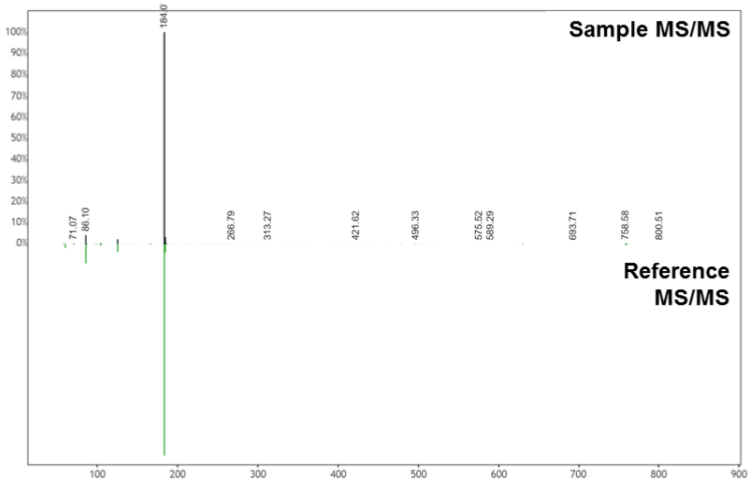

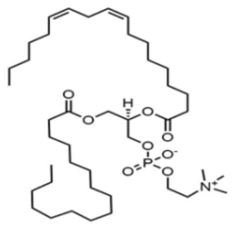

Spectral Match to PC(16:0/18:2)
Cosine score: 0.91
ppm error: 2.48

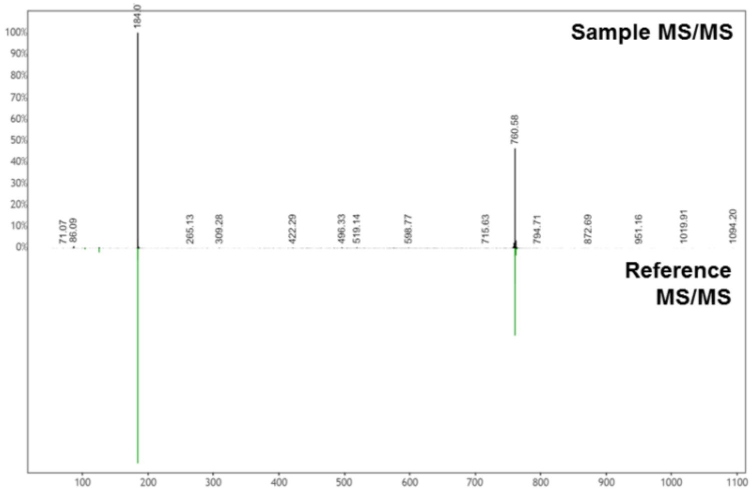

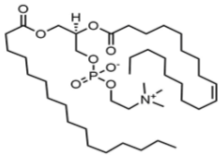

Spectral Match to PC(16:0/18:1)
Cosine score: 0.98
ppm error: 1.28

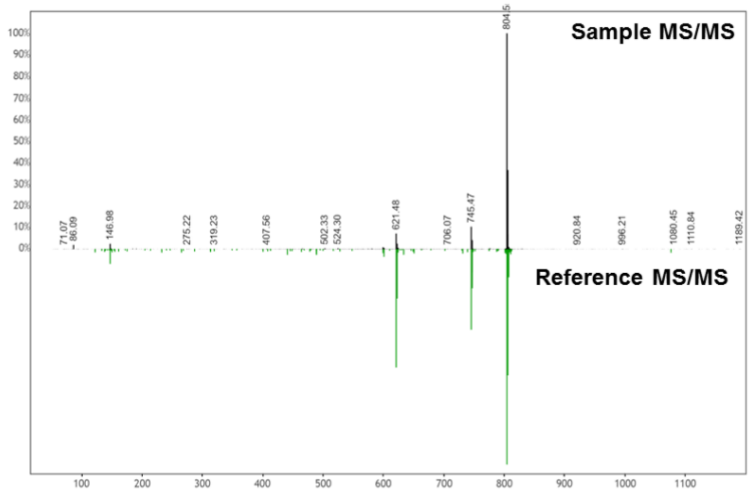

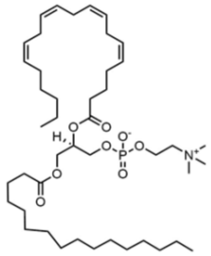

Spectral Match to PC(16:0/20:4)
Cosine score: 0.75
ppm error: 0.16

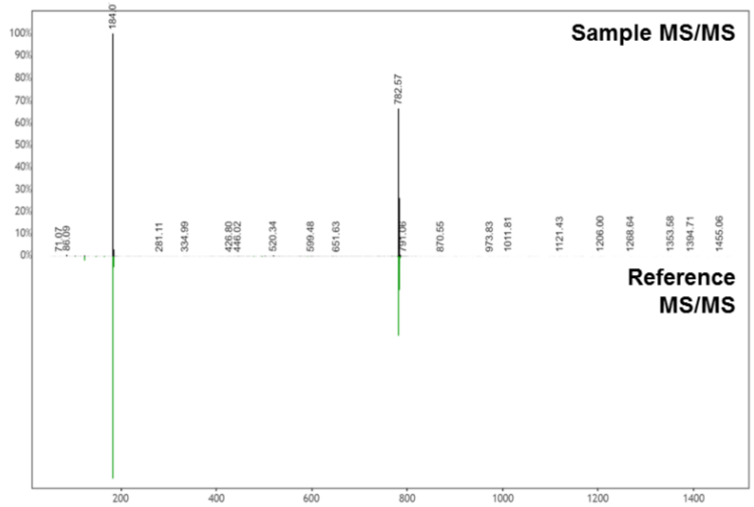

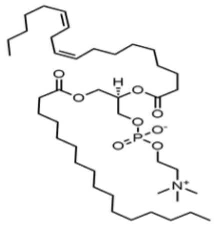

Spectral Match to PC(18:2/18:2)
Cosine score: 0.94
ppm error: 4.19
