# Supplementary material for: Small in size, big on taste: Metabolomics analysis of flavor compounds from Philippine garlic
Source: PLoS One. 2021 May 20;16(5):e0247289. doi: 10.1371/journal.pone.0247289 (PMC8136657; doi:10.1371/journal.pone.0247289)
Supplement: S5 Fig — (PDF) [file pone.0247289.s005.pdf]

S5. Multivariate Analysis via XCMS Online: Pairwise Data for ILAU and BAU

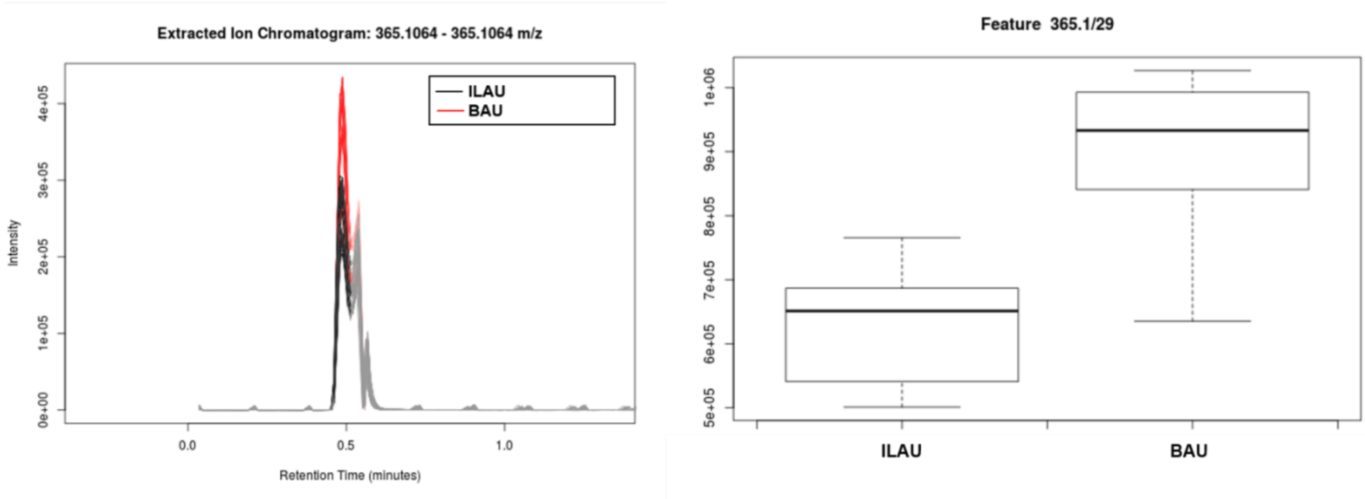

S5 Figure 1. Extracted ion chromatogram (left) for feature 365.1064, identified by GNPS as melibiose. Box-and-whisker representation (right) show that melibiose is upregulated in BAU samples and down regulated in ILAU samples.

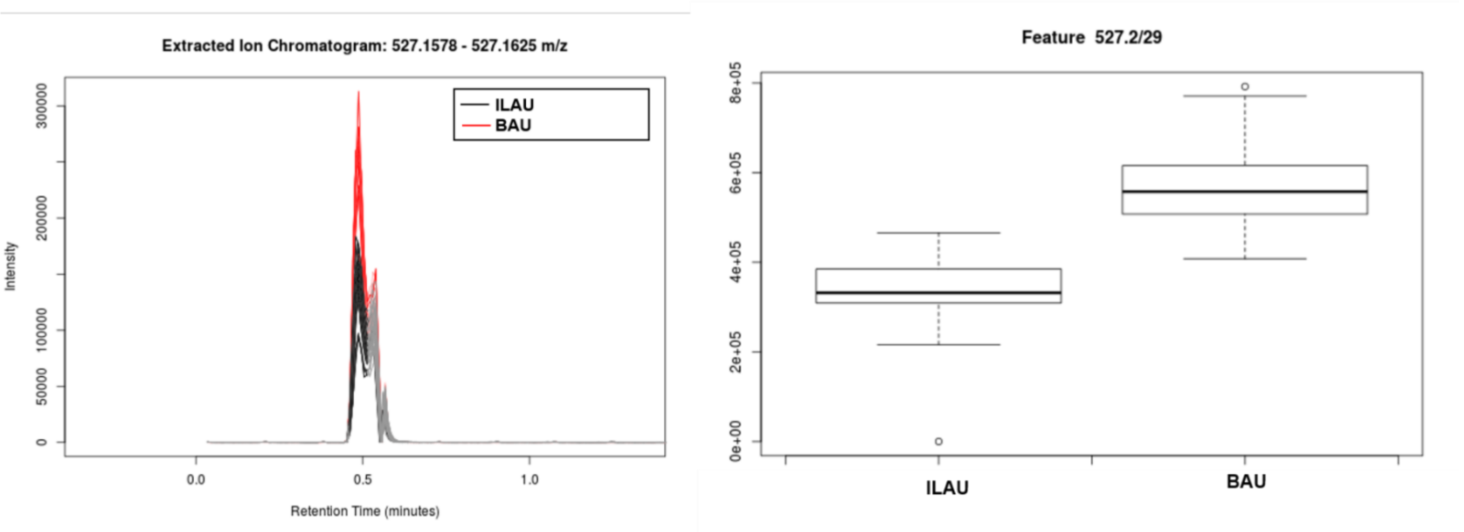

S5 Figure 2. Extracted ion chromatogram (left) for feature 527.1578, identified by GNPS as 1-kestose. Box-and-whisker representation (right) show that 1-kestose is present in higher abundance in BAU samples compared to ILAU.

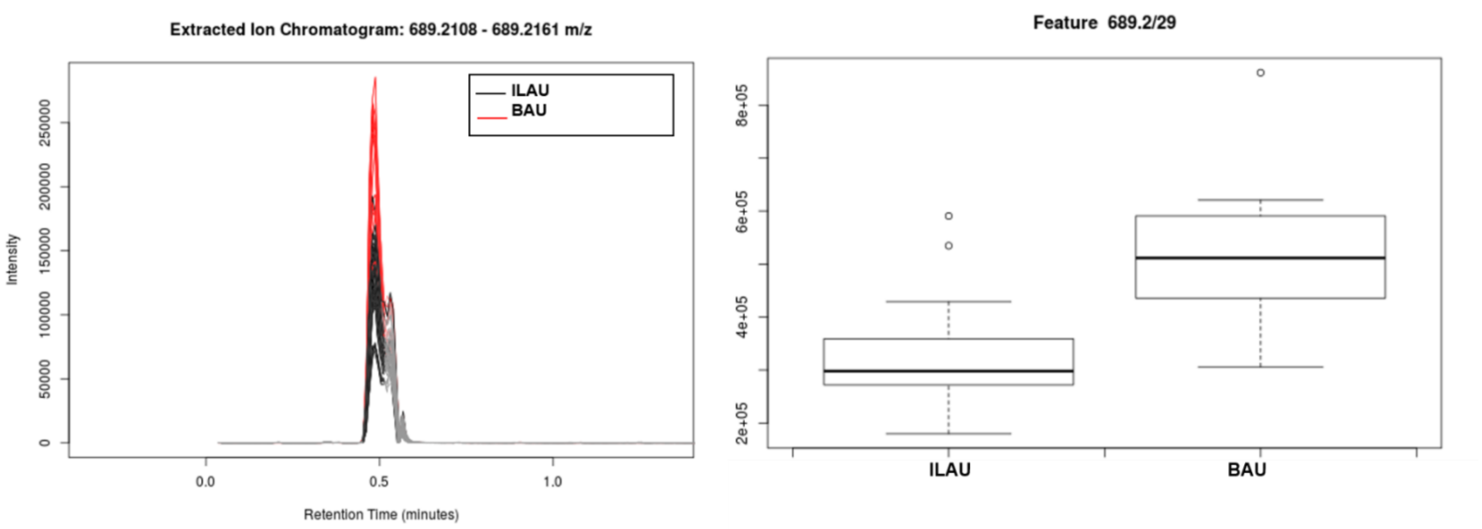

S5 Figure 3. Extracted ion chromatogram (left) for feature 689.2108, identified by GNPS as stachyose. Box-and-whisker representation (right) show that stachyose is present in higher abundance in BAU samples compared to ILAU.

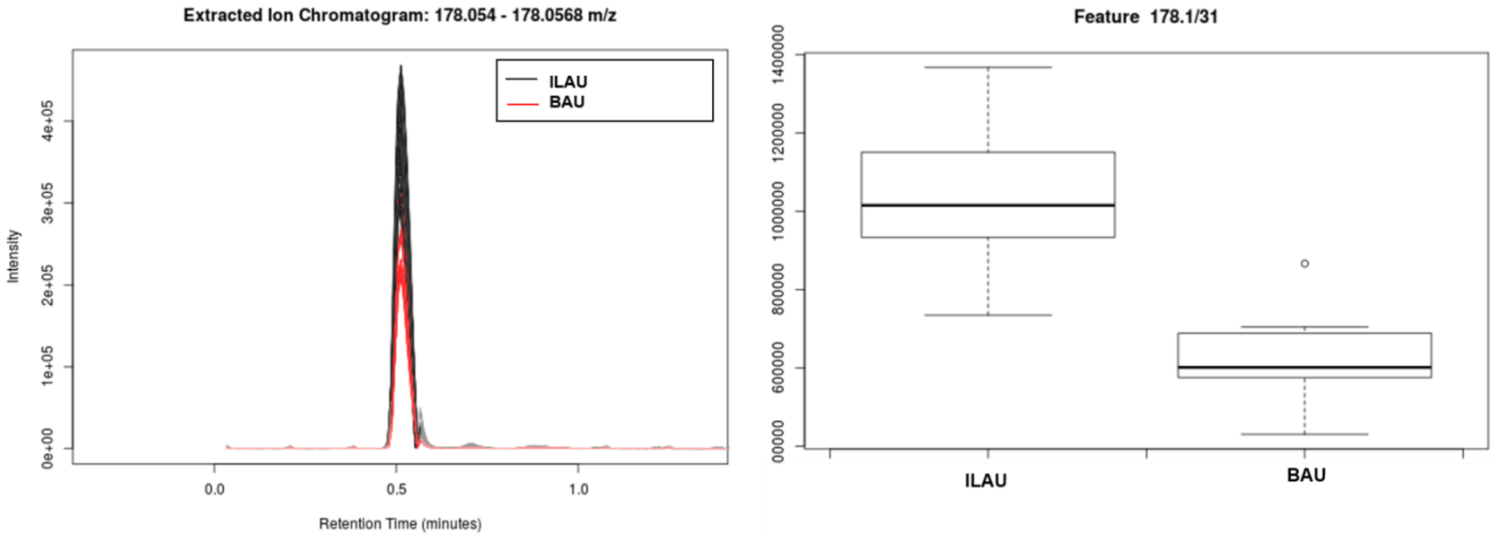

S5 Figure 4. Extracted ion chromatogram (left) for feature 178.0540, identified by GNPS as alliin. Box-and-whisker representation (right) show that alliin is present in higher abundance in ILAU samples compared to BAU.

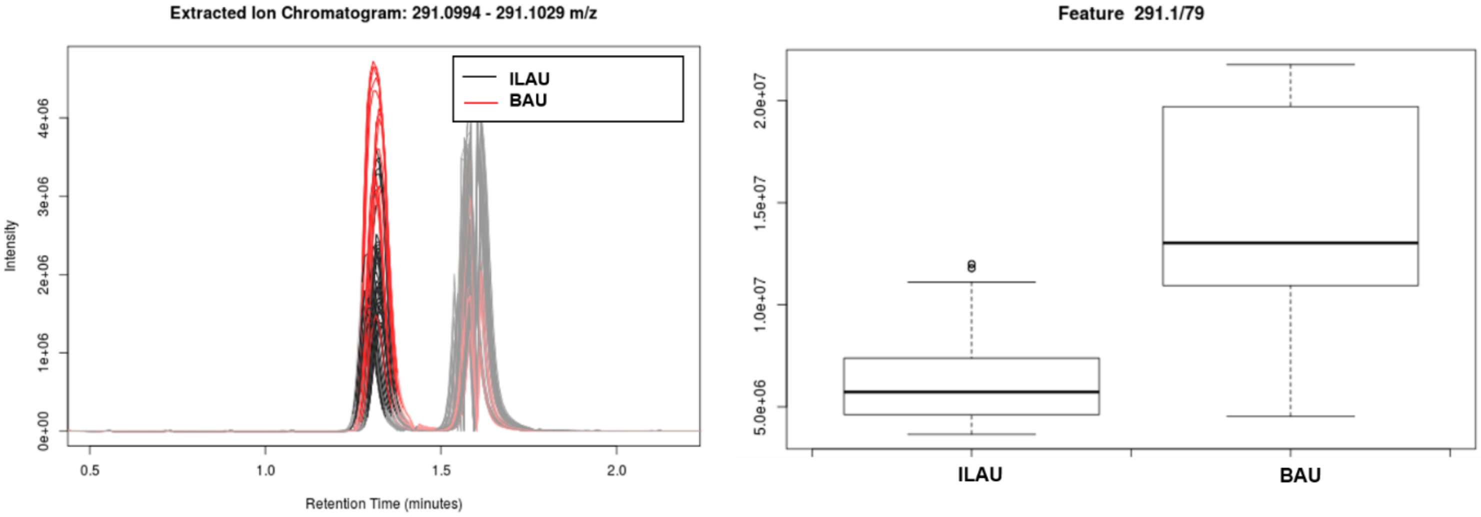

S5 Figure 5. Extracted ion chromatogram (left) for feature 291.1029 eluting at 1.316 minutes was manually identified as γ-glutamyl allyl cysteine. Box-and-whisker (right) representation shows that this compound is upregulated in BAU samples.

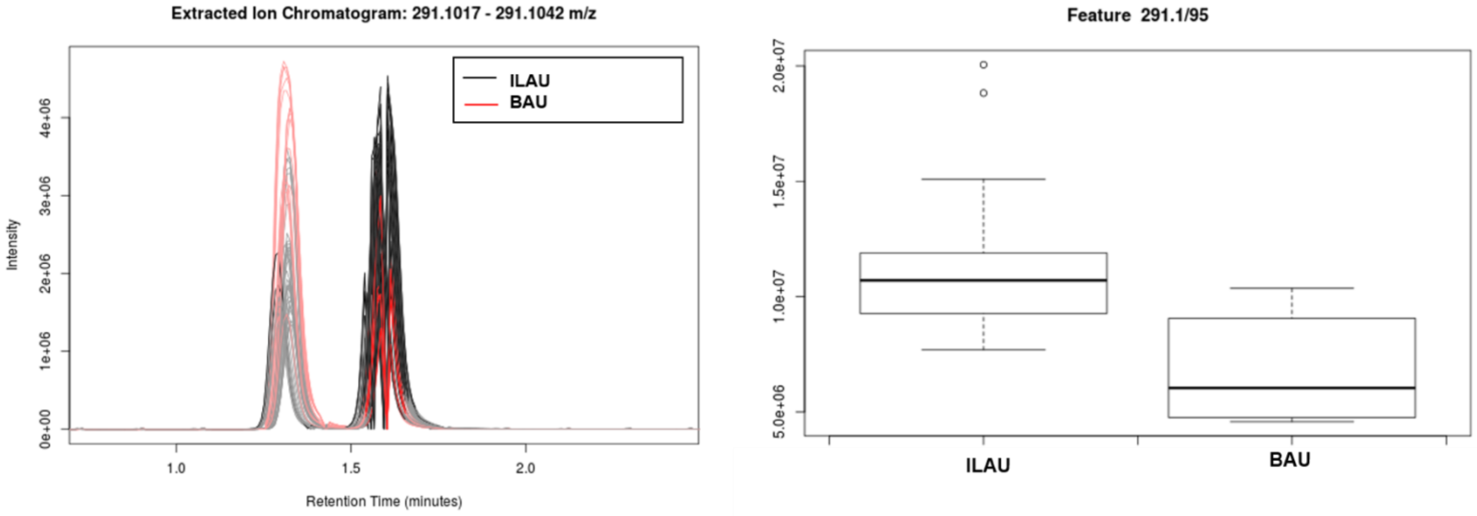

S5 Figure 6. Extracted ion chromatogram (left) for feature 291.1029 eluting at 1.584 minutes was manually identified as an isomer of γ-glutamyl allyl cysteine. Box-and-whisker (right) representation shows that this compound is downregulated in BAU samples and upregulated in ILAU.

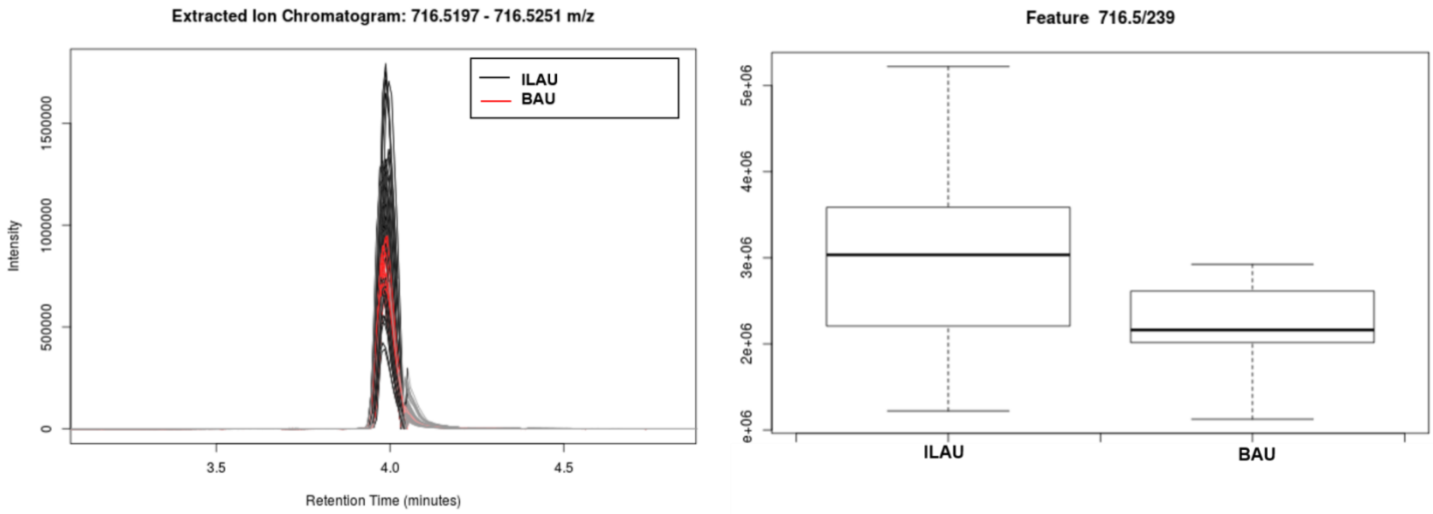

S5 Figure 7. Extracted ion chromatogram (left) for feature 716.5251 was identified by GNPS as a lipid derivative. Box-and-whisker (right) representation shows that this compound is present in both samples but is more abundant in ILAU.
