# Supplementary material for: Small in size, big on taste: Metabolomics analysis of flavor compounds from Philippine garlic
Source: PLoS One. 2021 May 20;16(5):e0247289. doi: 10.1371/journal.pone.0247289 (PMC8136657; doi:10.1371/journal.pone.0247289)
Supplement: S6 Fig — (PDF) [file pone.0247289.s006.pdf]

# S6. Multigroup comparison between ILAU, BAU, and LA

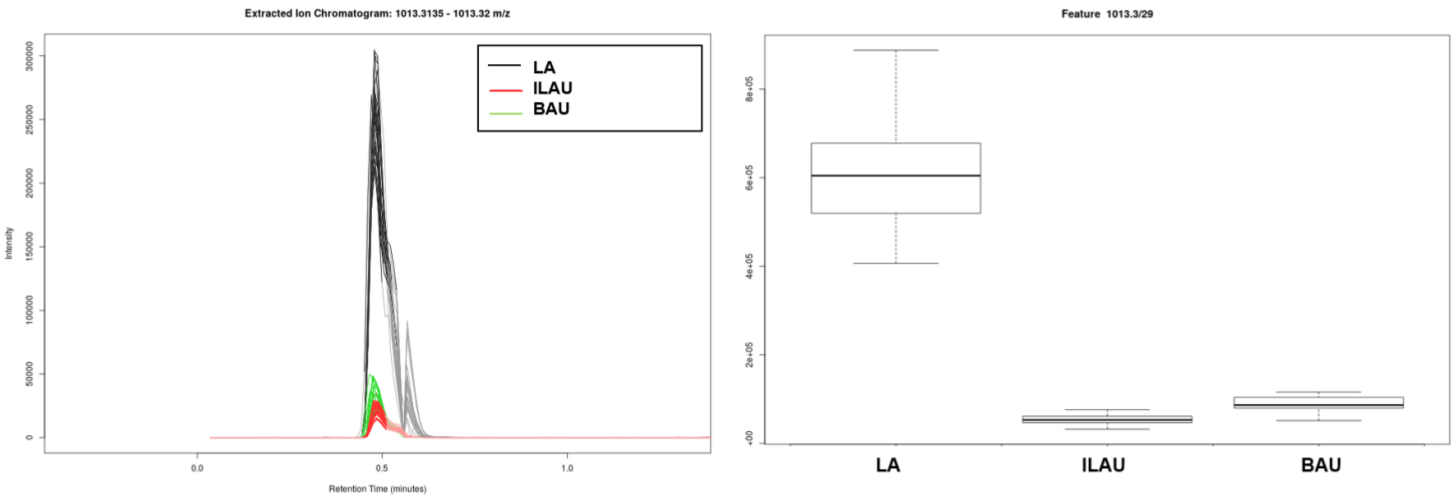


**S6 Figure 1. Extracted ion chromatogram (left) for feature 1013.3135, manually annotated via MS/MS as a hexaoligosaccharide.** Box-and-whisker (right) representation shows that this compound is highly upregulated in LA samples.

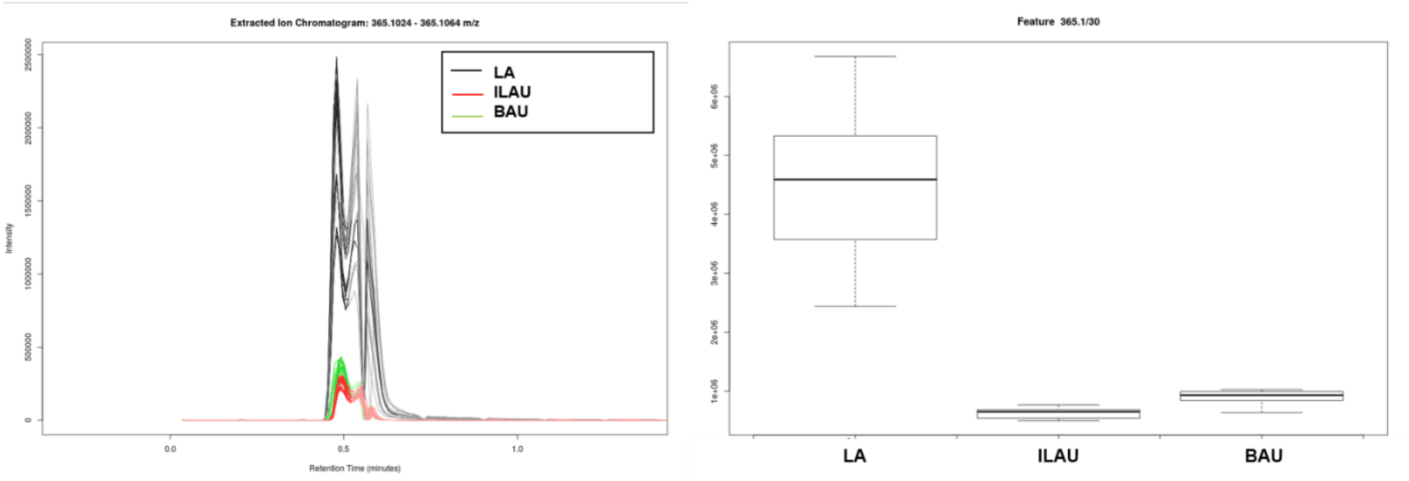


**S6 Figure 2. Extracted ion chromatogram (left) for feature 365.1064, annotated by GNPS as melibiose**. Box-and-whisker (right) representation shows that this compound is highly upregulated in LA samples.

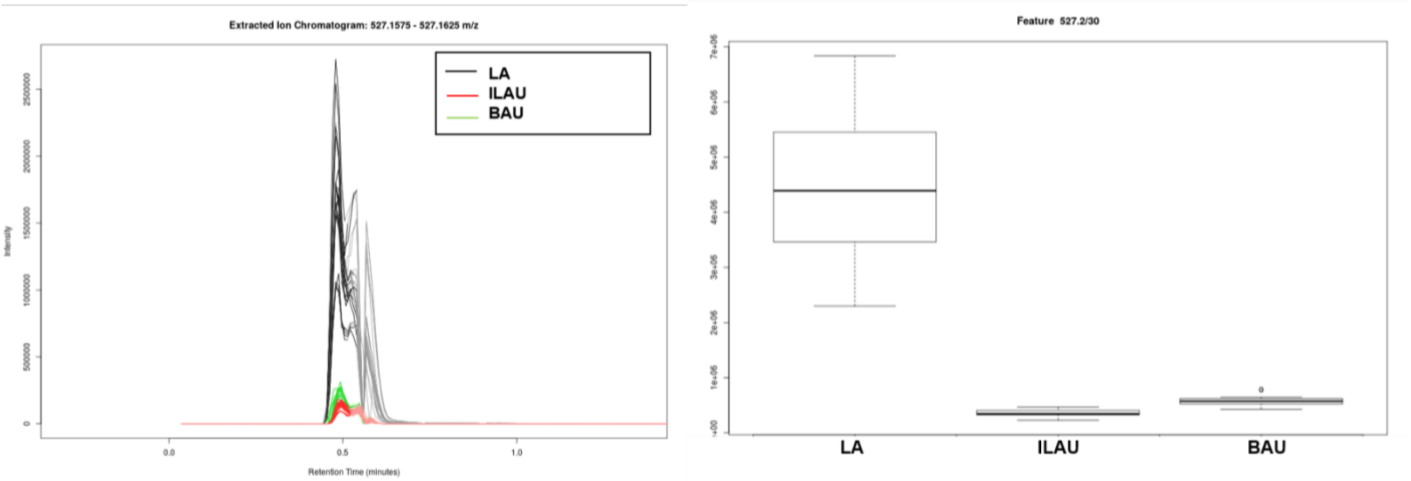


**S6 Figure 3. Extracted ion chromatogram (left) for feature 527.1575, annotated by GNPS as 1-kestose**. Box-and-whisker (right) representation shows that this compound is highly upregulated in LA samples.

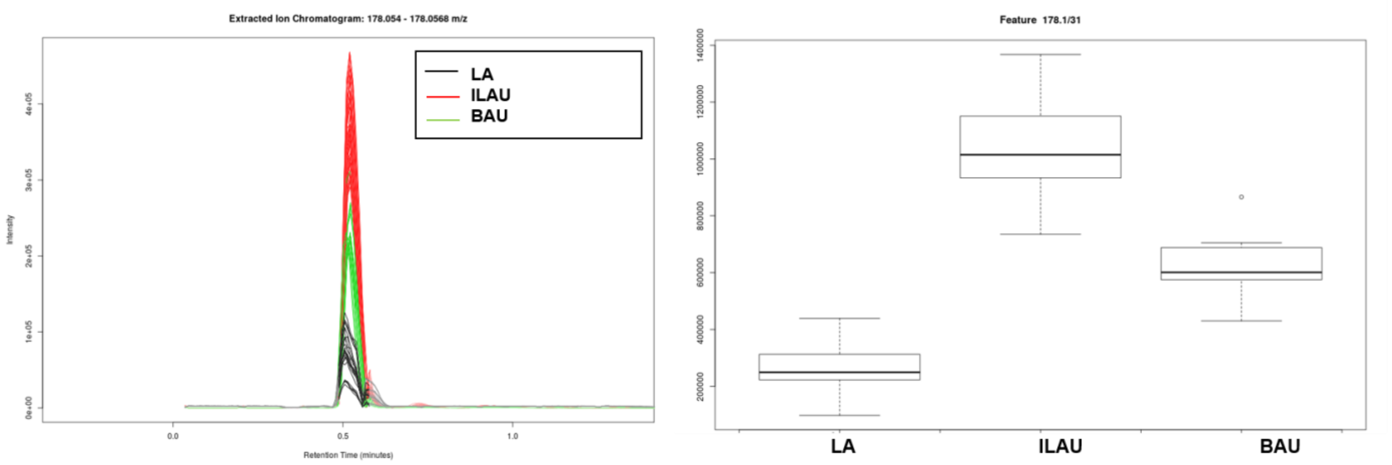


**S6 Figure 4. Extracted ion chromatogram (left) for feature 178.0540, annotated by GNPS as alliin.** Box-and-whisker (right) representation shows that this compound is highly upregulated in ILAU samples.

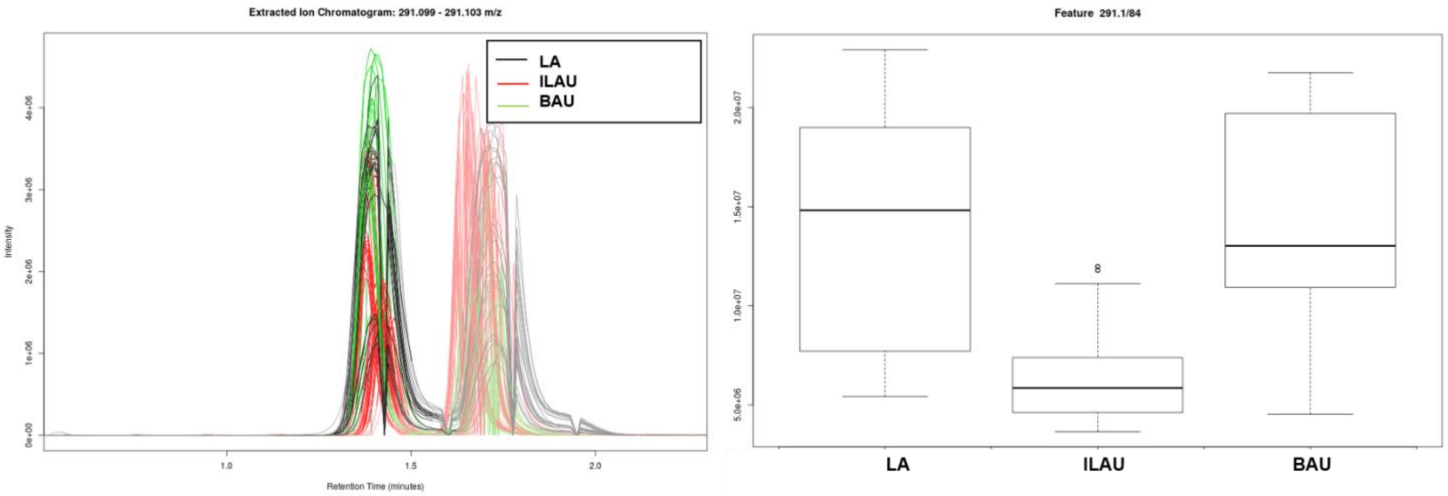


**S6 Figure 5. Extracted ion chromatogram (left) for feature 291.1029 eluting at 1.401 minutes was manually identified as γ-glutamyl allyl cysteine**. Box-and-whisker (right) representation shows that this compound is upregulated in BAU and LA samples.

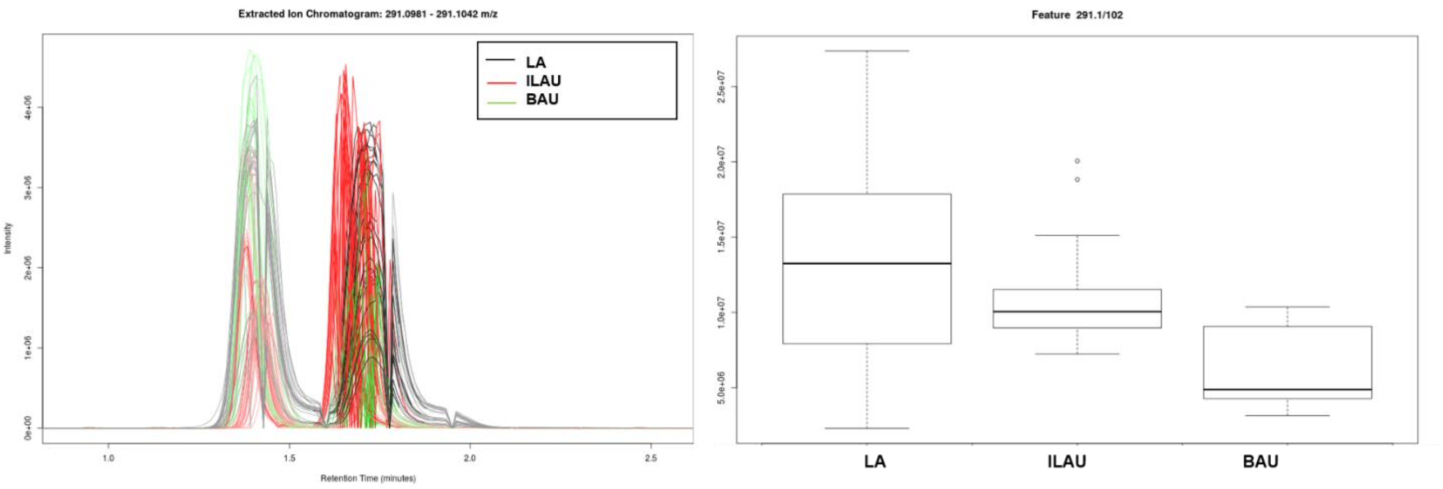


**S6 Figure 6. Extracted ion chromatogram (left) for feature 291.1029 eluting at 1.706 minutes was manually identified as an isomer of γ-glutamyl allyl cysteine**. Box-and-whisker (right) representation shows that this compound is highly abundant in LA samples.

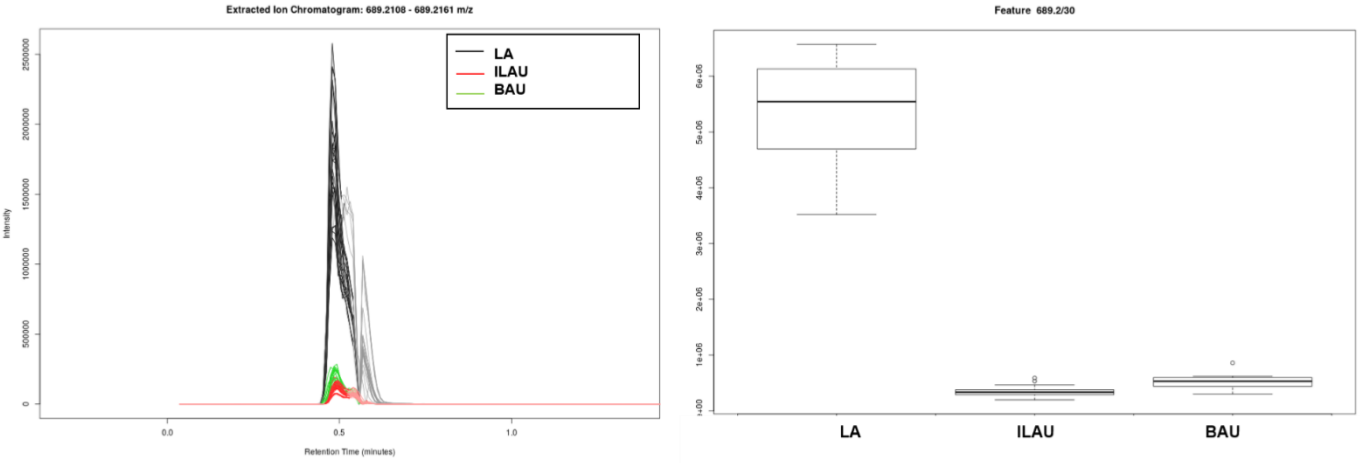


**S6 Figure 7. Extracted ion chromatogram (left) for feature 689.2108, annotated by GNPS as stachyose**. Box-and-whisker (right) representation shows that this compound is highly upregulated in LA samples.

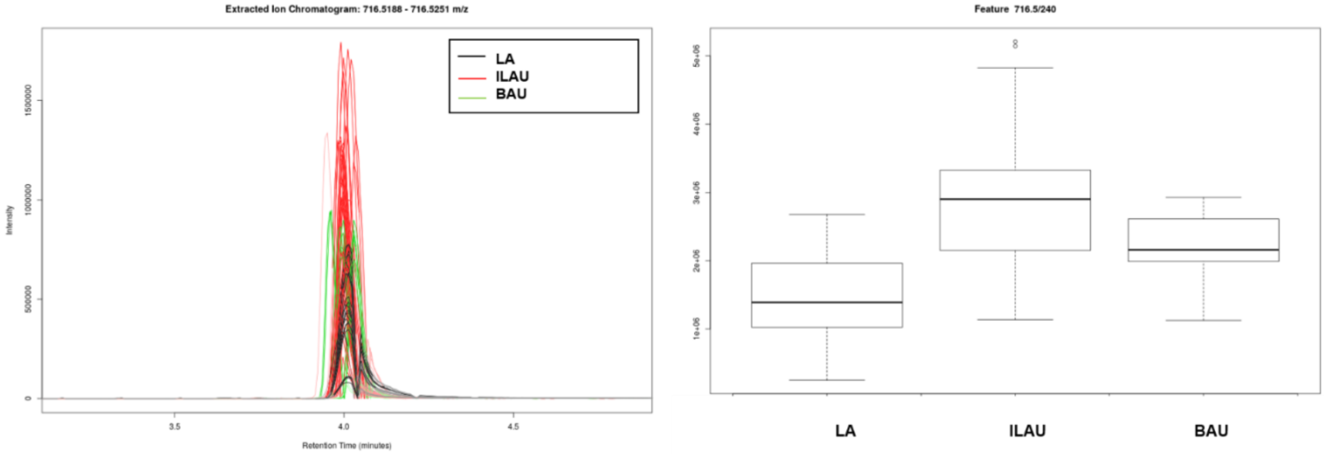


**S6 Figure 8. Extracted ion chromatogram (left) for feature 716.5251 was identified by GNPS as a lipid derivative.** Box-and-whisker (right) representation shows that this compound is present in all three samples but is more abundant in ILAU.
