# Supplementary material for: Small in size, big on taste: Metabolomics analysis of flavor compounds from Philippine garlic
Source: PLoS One. 2021 May 20;16(5):e0247289. doi: 10.1371/journal.pone.0247289 (PMC8136657; doi:10.1371/journal.pone.0247289)
Supplement: S7 Fig — (PDF) [file pone.0247289.s007.pdf]

S7. Multigroup analysis of known local samples

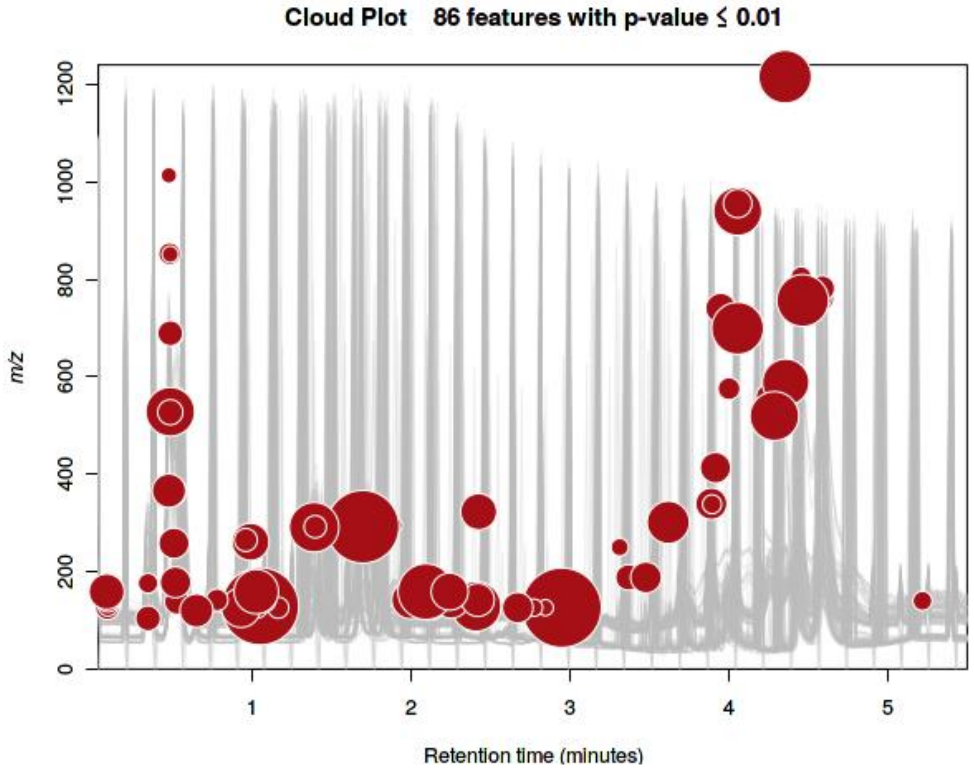

S7 Figure 1. **XCMS cloud plot of all known local samples**. Fold change corresponds proportionally to the radius of the circle.

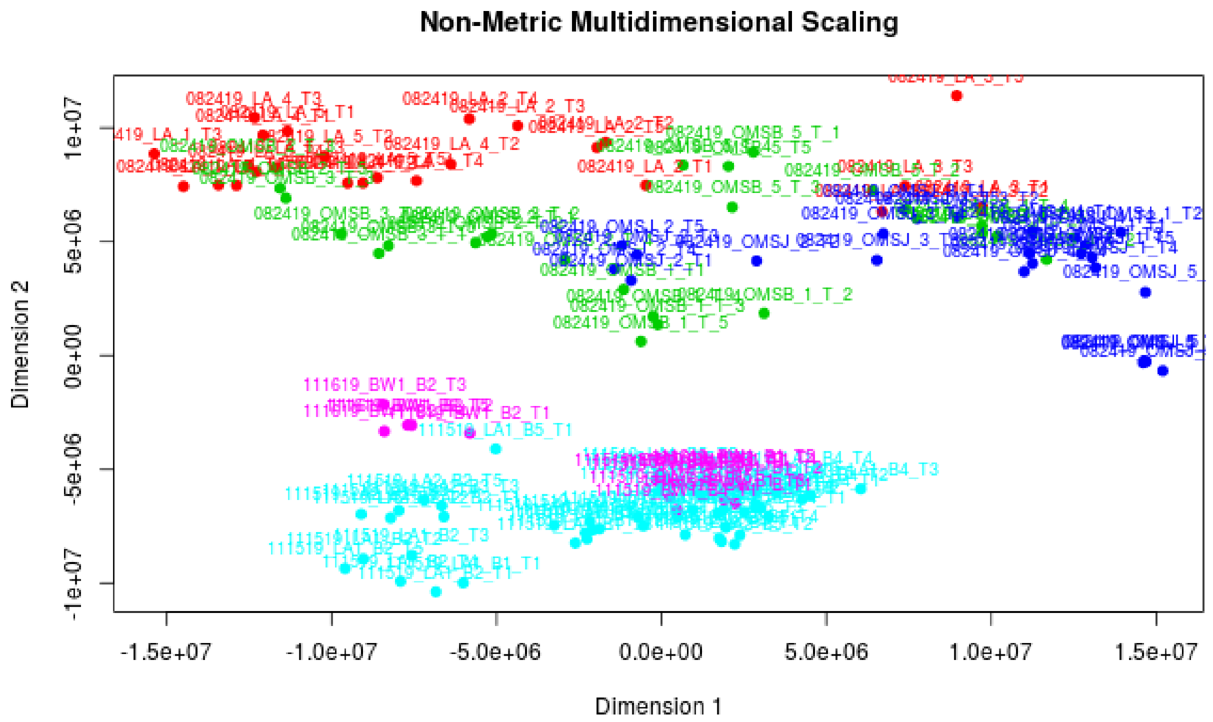

S7 Figure 2. NMDS biplot of known local samples. This show close similarities between benchmark samples ILAU and BAU (light blue and purple), which are distinct from the rest of the samples consisting of LA, OMSB, and OMSJ. Majority of LA samples also share features with that of OMSB than OMSJ.

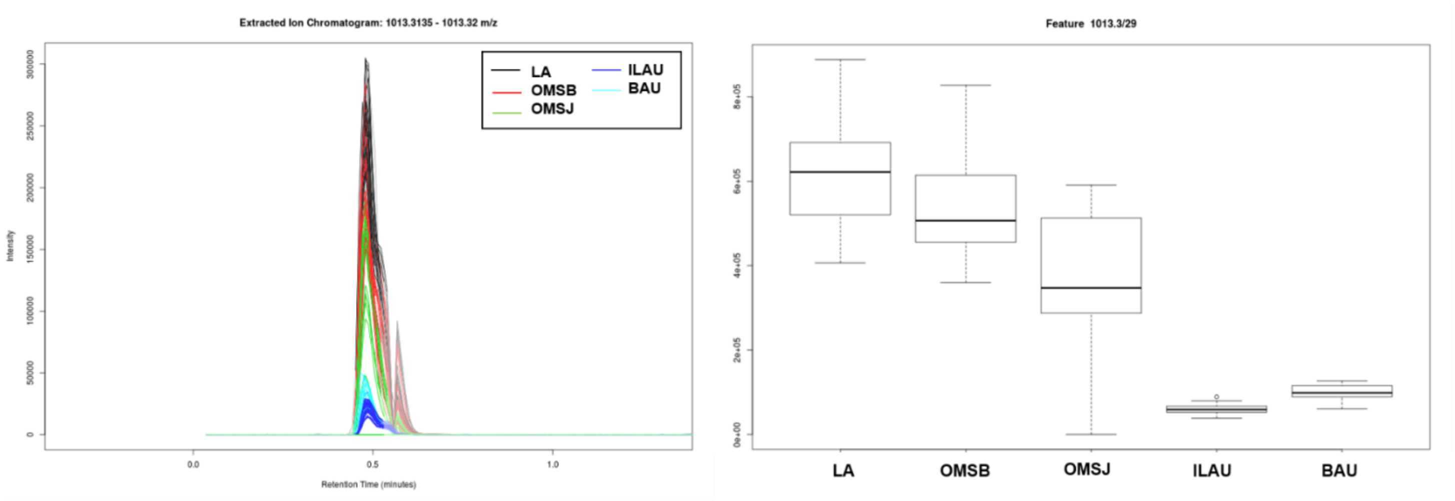

S7 Figure 3. Extracted ion chromatogram (left) for feature 1013.3135, manually annotated via MS/MS as a hexaoligosaccharide. Box-and-whisker (right) representation shows that this compound is highly upregulated in LA, OMSB, and OMSJ samples, while it is downregulated in ILAU and BAU samples.

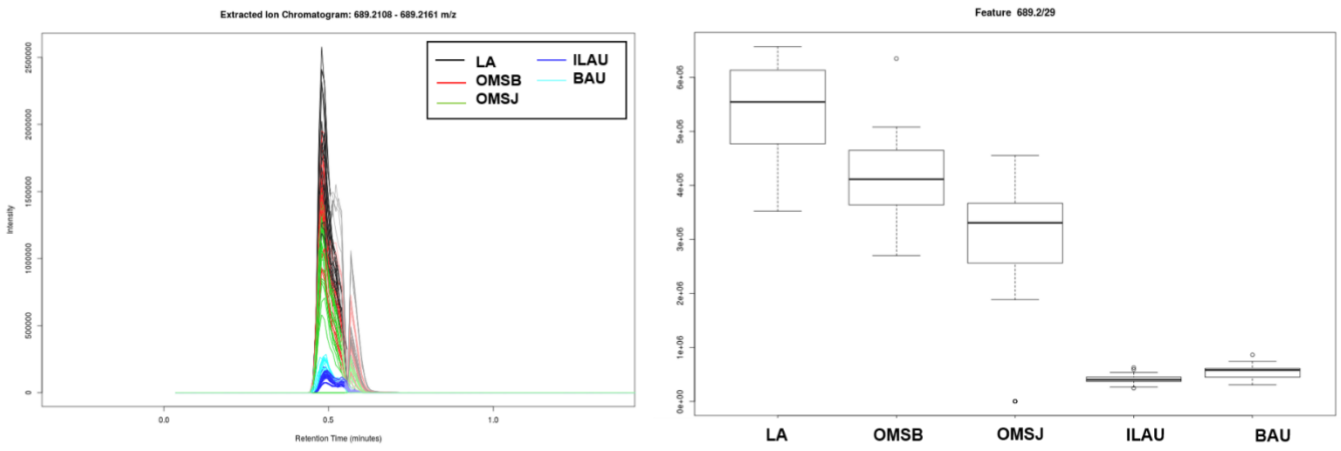

S7 Figure 4. Extracted ion chromatogram (left) for feature 689.2108, identified by GNPS as stachyose. Box-and-whisker representation (right) show that stachyose is present in higher abundance in non-authenticated samples.

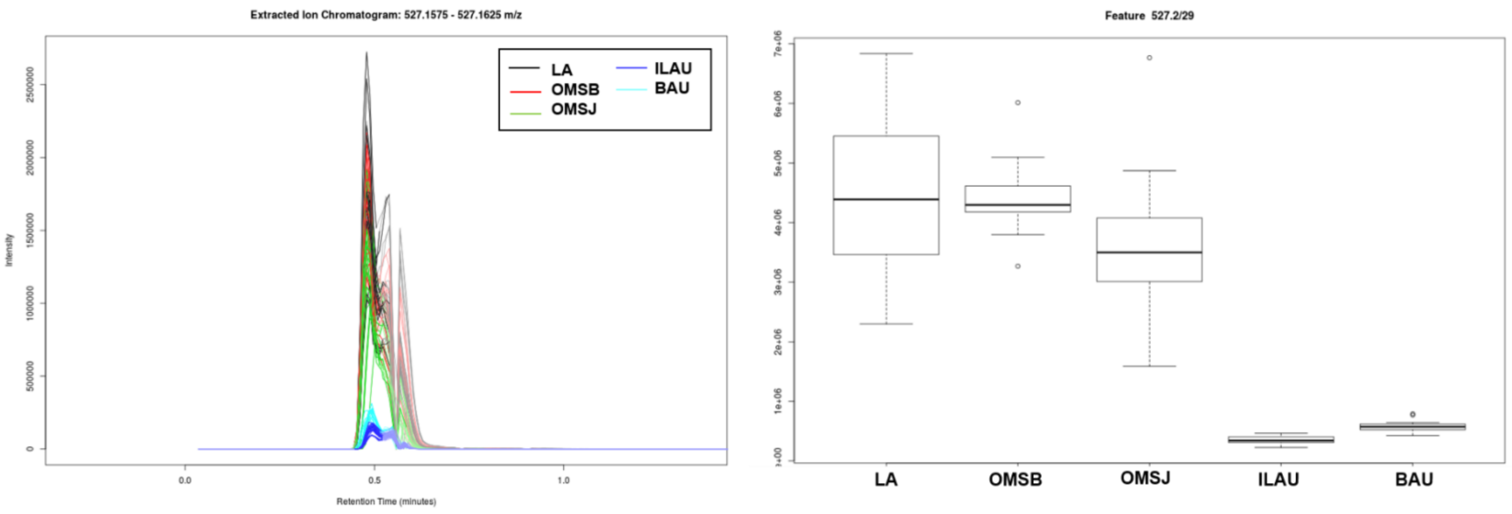

S7 Figure 5. Extracted ion chromatogram (left) for feature 527.1575, annotated by GNPS as 1-kestose. Box-and-whisker (right) representation shows that this compound is highly downregulated in benchmark samples.

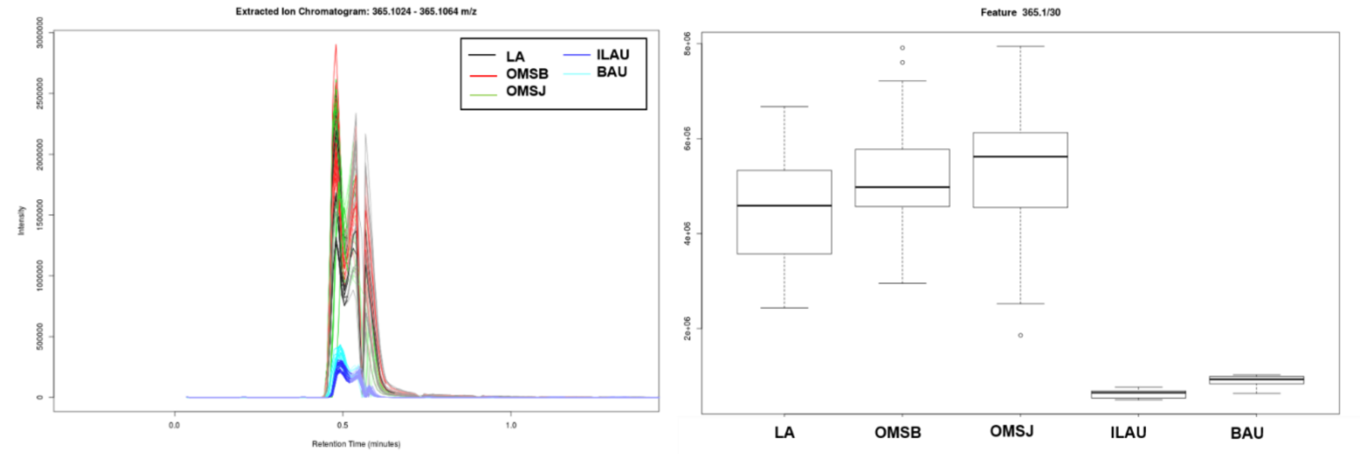

S7 Figure 6. Extracted ion chromatogram (left) for feature 365.1064, annotated by GNPS as melibiose. Box-and-whisker (right) representation shows that this compound is highly downregulated in benchmark samples

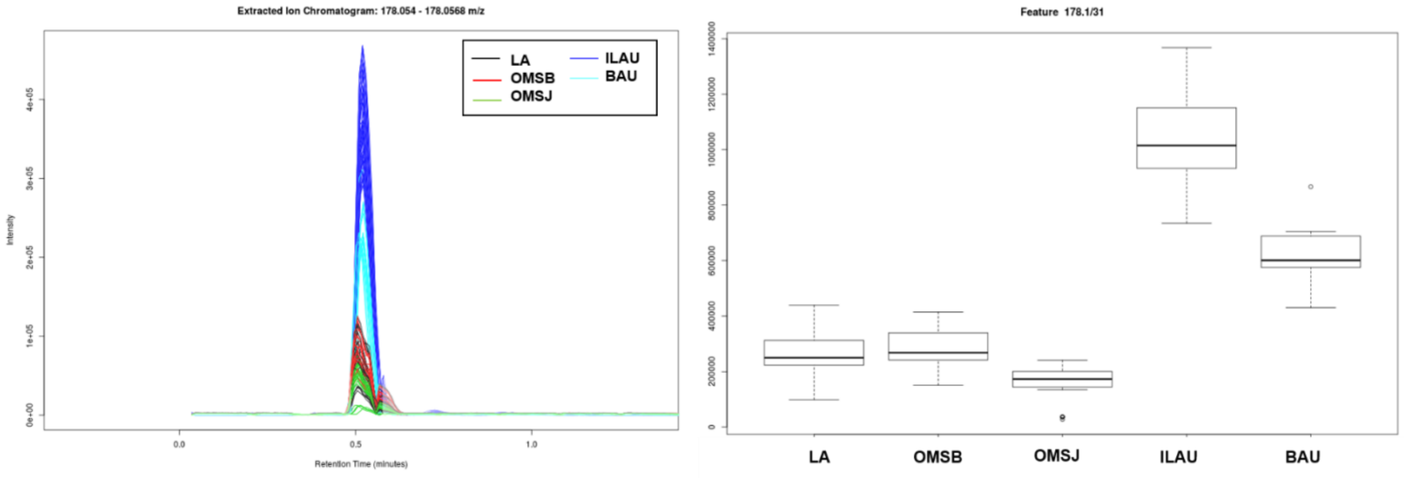

S7 Figure 7. Extracted ion chromatogram (left) for feature 178.0540, annotated by GNPS as alliin. Box-and-whisker (right) representation shows that this compound is highly upregulated in ILAU samples, followed by BAU.

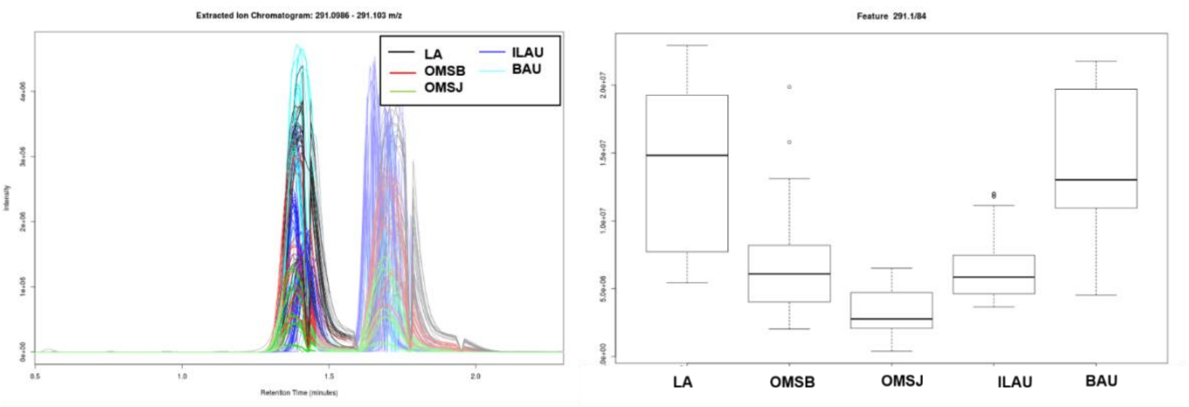

S7 Figure 8. Extracted ion chromatogram (left) for feature 291.1029 eluting at 1.401 minutes was manually identified as γ-glutamyl allyl cysteine. Box-and-whisker (right) representation shows that this compound is upregulated in BAU and LA samples.

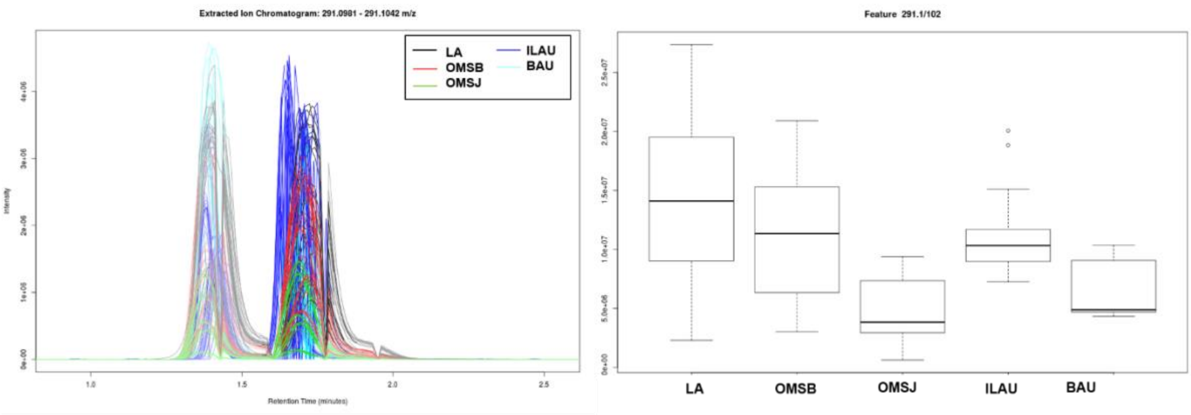

S7 Figure 9. Extracted ion chromatogram (left) for feature 291.1029 eluting at 1.706 minutes was manually identified as an isomer of γ-glutamyl allyl cysteine. Box-and-whisker (right) representation shows that this compound is highly abundant in LA samples.

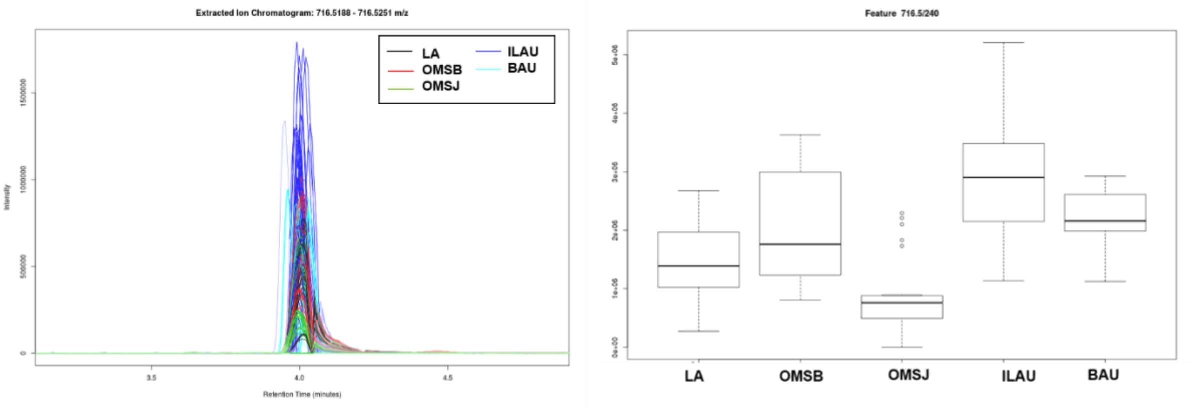

S7 Figure 10. Extracted ion chromatogram (left) for feature 716.5251 was identified by GNPS as a lipid derivative. Box-and-whisker (right) representation shows that this compound is present in all samples but is seen in higher abundance in ILAU.
