# Supplementary material for: Small in size, big on taste: Metabolomics analysis of flavor compounds from Philippine garlic
Source: PLoS One. 2021 May 20;16(5):e0247289. doi: 10.1371/journal.pone.0247289 (PMC8136657; doi:10.1371/journal.pone.0247289)
Supplement: S8 Fig — (PDF) [file pone.0247289.s008.pdf]

S8: Multigroup analysis of all known local and imported samples

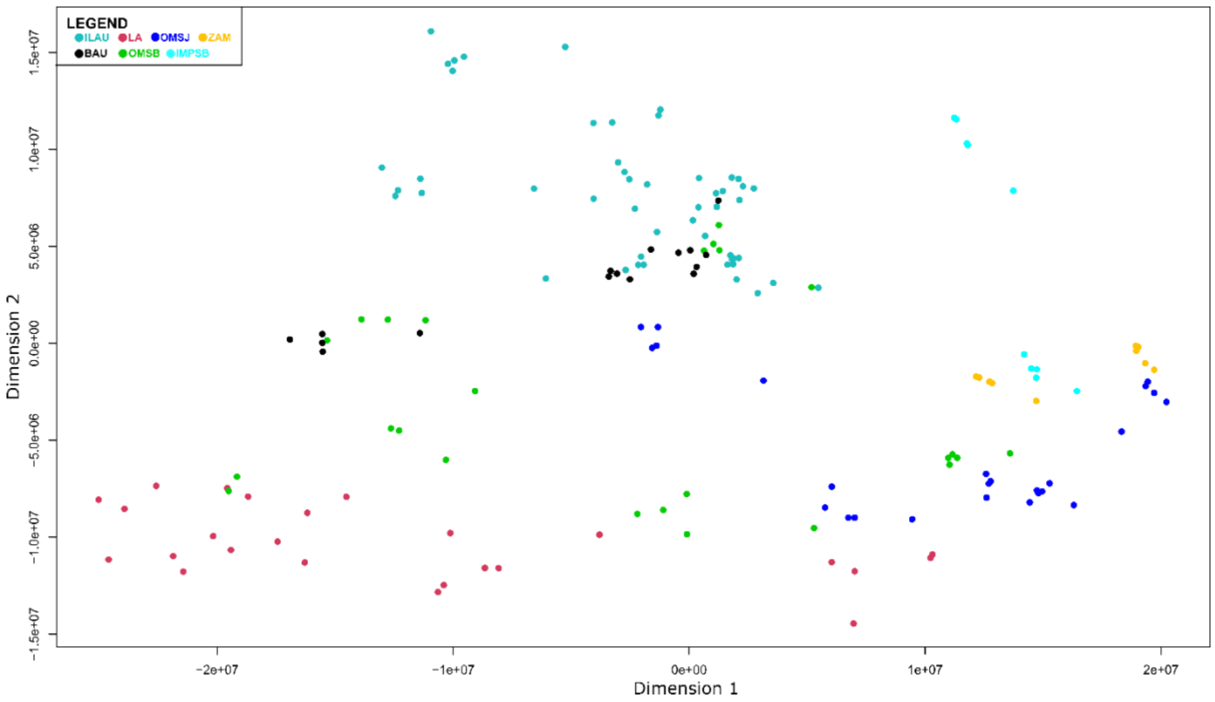

S8 Figure 1. NMDS analysis of known local and imported samples. ILAU and BAU samples share features with some OMSB and OMSJ samples, while LA have common features with OMSJ and OMSB but not with benchmarks samples. Imported samples ZAM and IMPSB separate from benchmark samples but have common features with majority of the OMSJ.

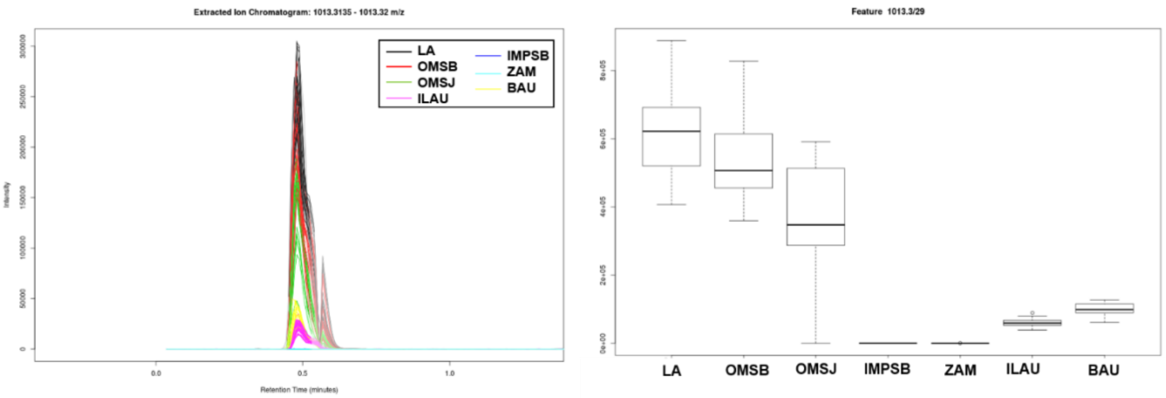

S8 Figure 2. Extracted ion chromatogram (left) for feature 1013.3135, manually annotated via MS/MS as a hexaoligosaccharide. Box-and-whisker (right) representation shows that this compound is highly upregulated in LA, OMSB, and OMSJ samples, while it is highly downregulated in imported IMPSB and ZAM.

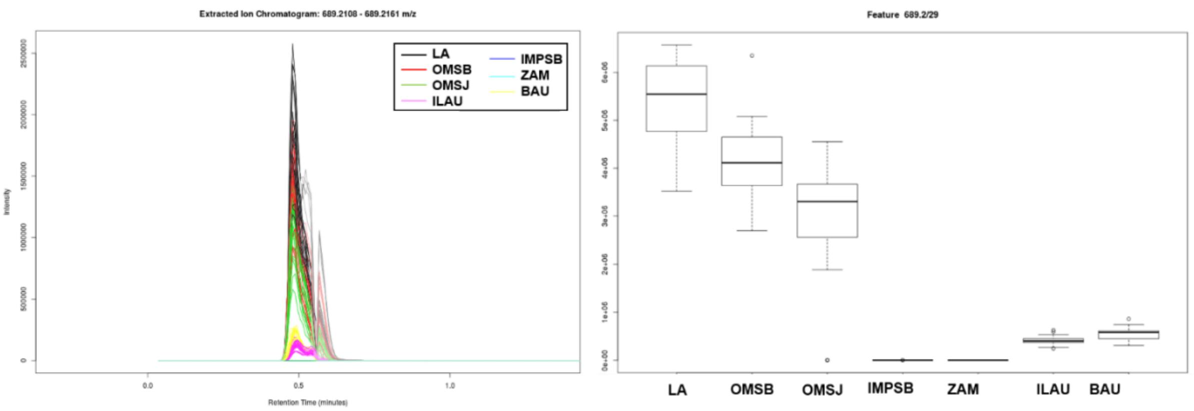

S8 Figure 3. Extracted ion chromatogram (left) for feature 689.2108, identified by GNPS as stachyose. Box-and-whisker representation (right) show that stachyose is present in higher abundance in non-authenticated local samples and is not present in IMPSB and ZAM.

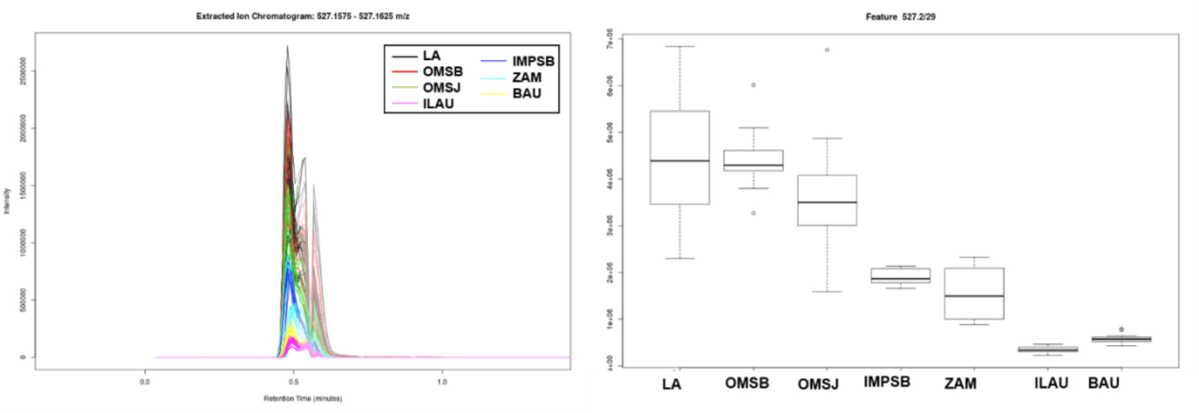

S8 Figure 4. Extracted ion chromatogram (left) for feature 527.1575, annotated by GNPS as 1-kestose. Box-and-whisker (right) representation shows that this compound is highly downregulated in benchmark samples.

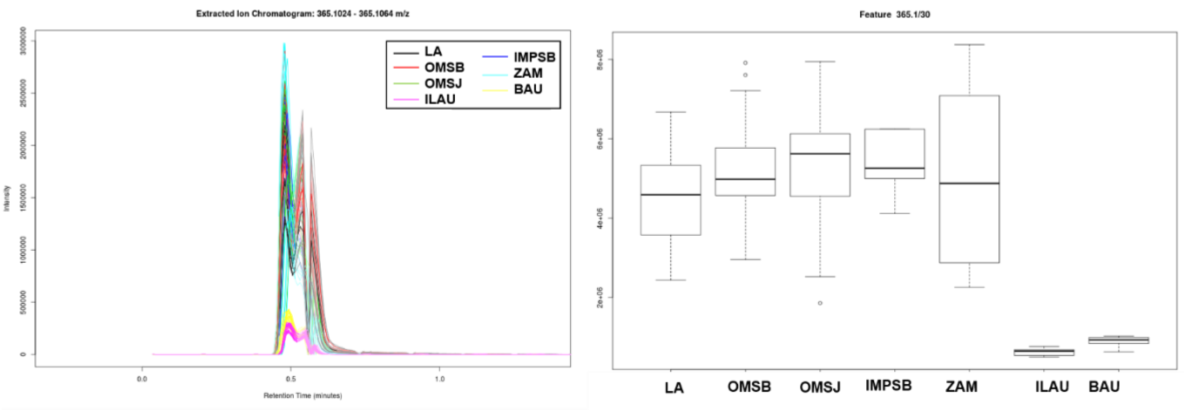

S8 Figure 5. Extracted ion chromatogram (left) for feature 365.1064, annotated by GNPS as melibiose. Box-and-whisker (right) representation shows that this compound is highly downregulated in benchmark samples and is a shared feature in LA, OMSB, OMSJ, and the imported bulbs IMPSB and ZAM.

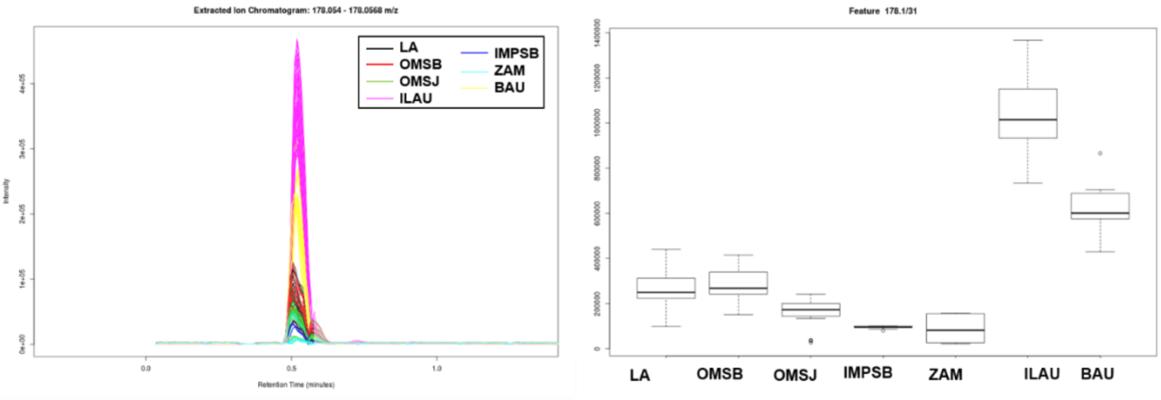

S8. Figure 6. Extracted ion chromatogram (left) for feature 178.0540, annotated by GNPS as alliin. Box-and-whisker (right) representation shows that this compound is highly upregulated in ILAU samples, followed by BAU. This feature is also present in low abundance in imported varieties.

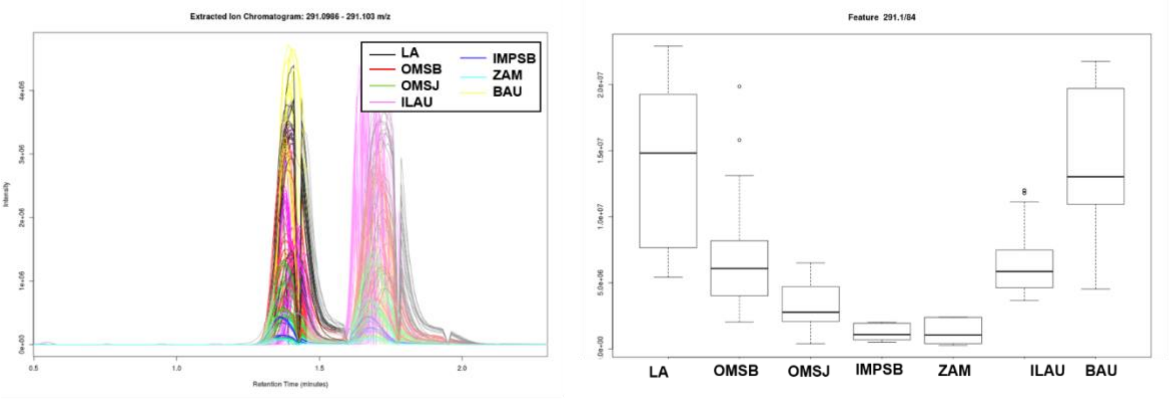

S8 Figure 7. Extracted ion chromatogram (left) for feature 291.1029 eluting at 1.401 minutes was manually identified as γ-glutamyl allyl cysteine. Box-and-whisker (right) representation shows that this compound is upregulated in BAU and LA samples and downregulated in IMPSB and ZAM.

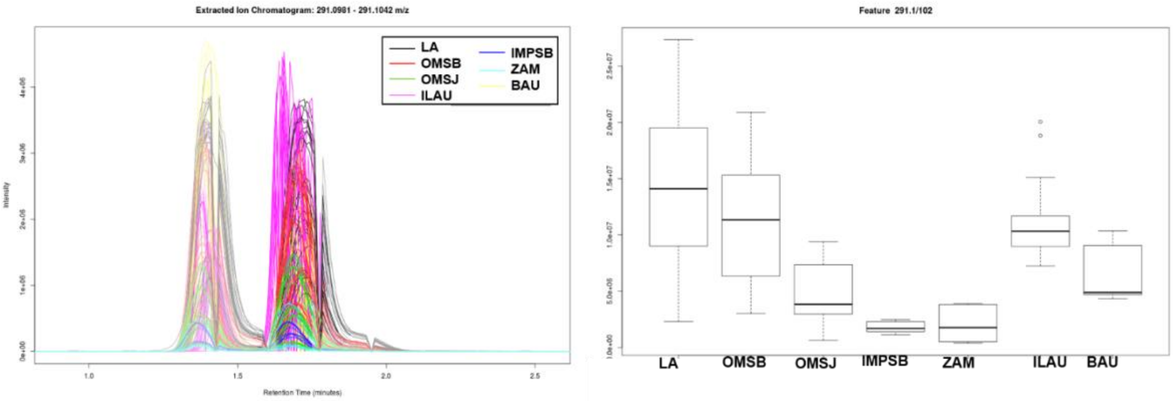

S8 Figure 8. Extracted ion chromatogram (left) for feature 291.1029 eluting at 1.706 minutes was manually identified as an isomer of γ-glutamyl allyl cysteine. Box-and-whisker (right) representation shows that this compound is highly abundant in LA samples.

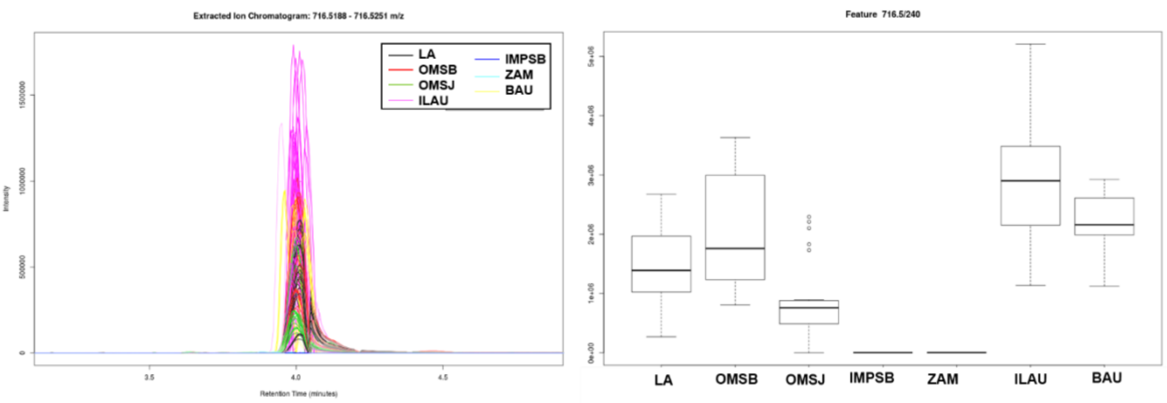

S8 Figure 9. Extracted ion chromatogram (left) for feature 716.5251 was identified by GNPS as a lipid derivative. Box-and-whisker (right) representation shows that this compound is present in all local samples, but not in imported garlic.
