# Supplementary material for: Small in size, big on taste: Metabolomics analysis of flavor compounds from Philippine garlic
Source: PLoS One. 2021 May 20;16(5):e0247289. doi: 10.1371/journal.pone.0247289 (PMC8136657; doi:10.1371/journal.pone.0247289)
Supplement: S9 Fig — (PDF) [file pone.0247289.s009.pdf]

S9: Multigroup analysis of samples with known origins vs unknown

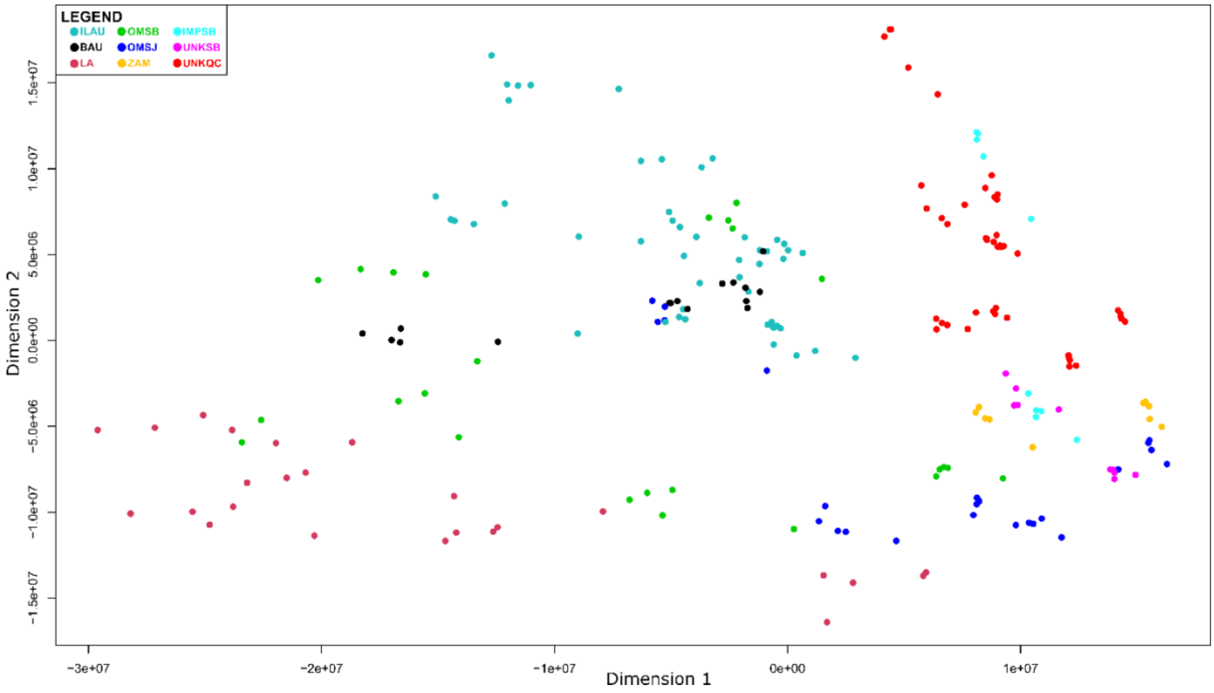

S9 Figure 1. NMDS analysis of known local and imported samples versus unknown. Distinct separation is seen between benchmark local samples and UNKQC and IMPSB, which in turn share features.

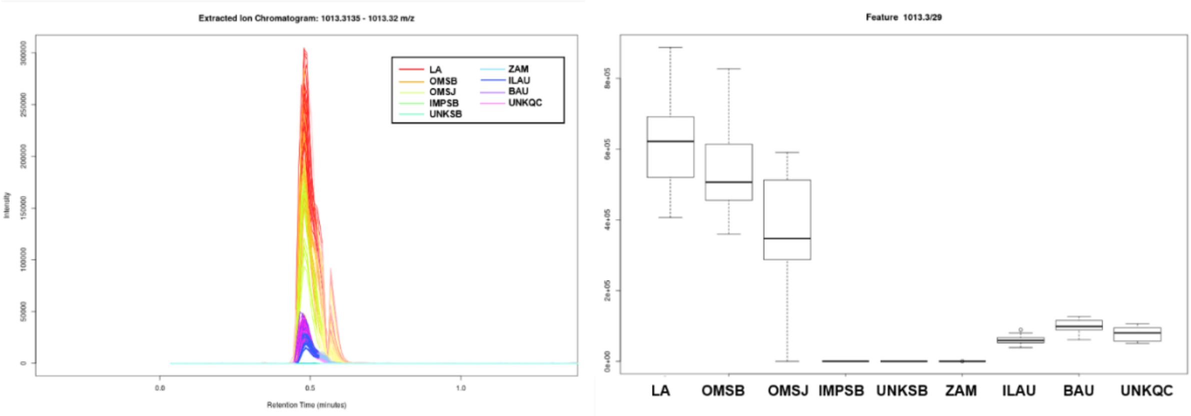

S9 Figure 2. Extracted ion chromatogram (left) for feature 1013.3135, manually annotated via MS/MS as a hexaoligosaccharide. Box-and-whisker (right) representation shows that this compound is highly upregulated in LA, OMSB, and OMSJ samples, while it may not be present in significant abundance in imported IMPSB, UNKSB, and ZAM.

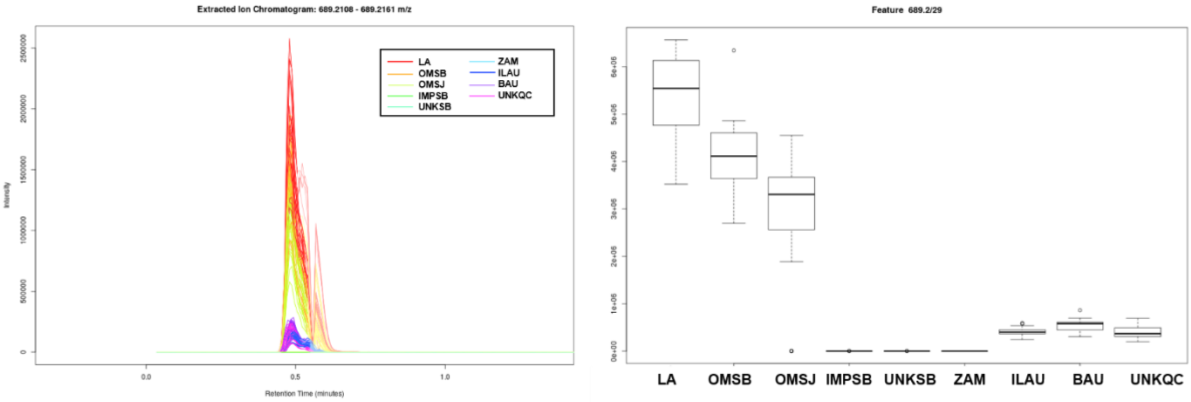

S9 Figure 3. Extracted ion chromatogram (left) for feature 689.2108, identified by GNPS as stachyose. Box-and-whisker representation (right) show that stachyose is present in higher abundance in non-authenticated local samples and is not present in ZAM, IMPSB and UNKSB.

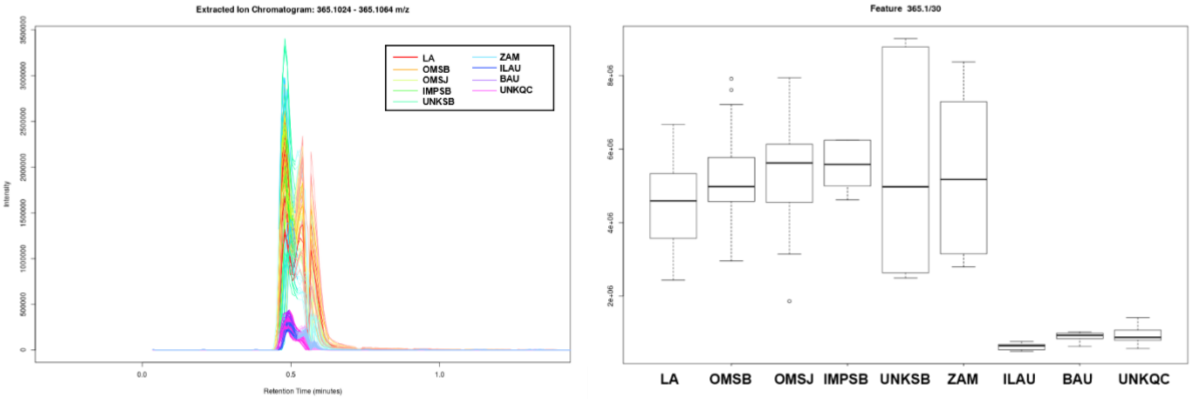

S9 Figure 4. Extracted ion chromatogram (left) for feature 365.1064, annotated by GNPS as melibiose. Box-and-whisker (right) representation shows that this compound is highly downregulated in benchmark samples and UNKQC and is a shared feature in LA, OMSB, OMSJ, and the imported bulbs IMPSB and ZAM, including UNKSB.

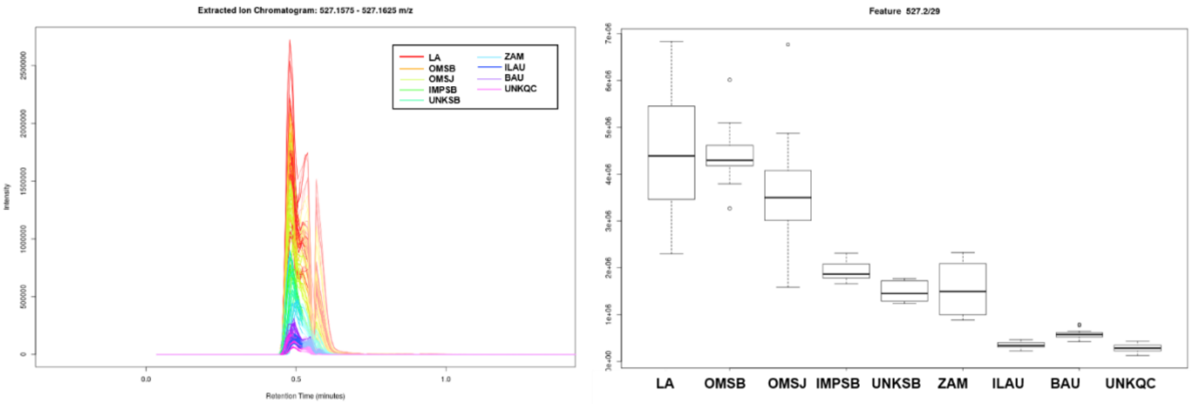

S9 Figure 5. Extracted ion chromatogram (left) for feature 527.1575, annotated by GNPS as 1-kestose. Box-and-whisker (right) representation shows that this compound is highly downregulated in benchmark samples and UNKQC.

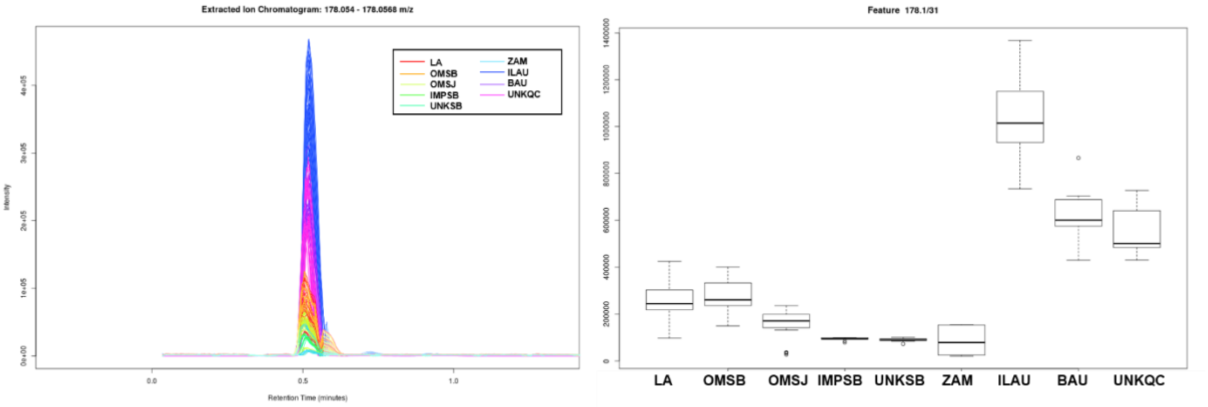

S9 Figure 6. Extracted ion chromatogram (left) for feature 178.0540, annotated by GNPS as alliin. Box-and-whisker (right) representation shows that this compound is highly upregulated in ILAU samples, followed by BAU and UNKQC. This feature is also present in low abundance in imported varieties IMPSB, ZAM, and UNKSB.

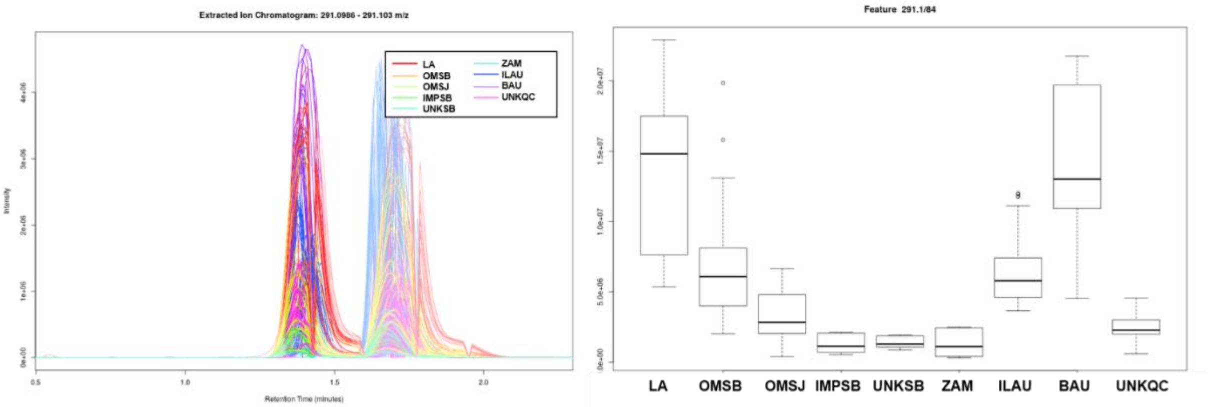

S9 Figure 7. Extracted ion chromatogram (left) for feature 291.1029 eluting at 1.401 minutes was manually identified as γ-glutamyl ally cysteine. Box-and-whisker (right) representation shows that this compound is upregulated in BAU and LA samples and downregulated in imported IMPSB and ZAB, and the unknown UNKSB and UNKQC.

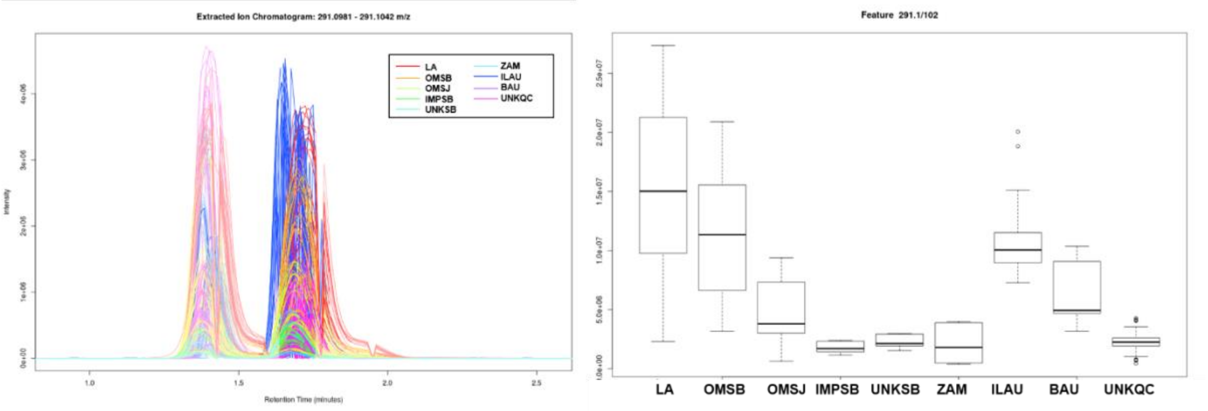

S9 Figure 8. Extracted ion chromatogram (left) for feature 291.1029 eluting at 1.706 minutes was manually identified as an isomer of γ-glutamyl ally cysteine. Box-and-whisker (right) representation shows that this compound is highly abundant in LA samples followed by OMSB and OMSJ. This feature is also downregulated in IMPSB, UNKSB and UNKQC.

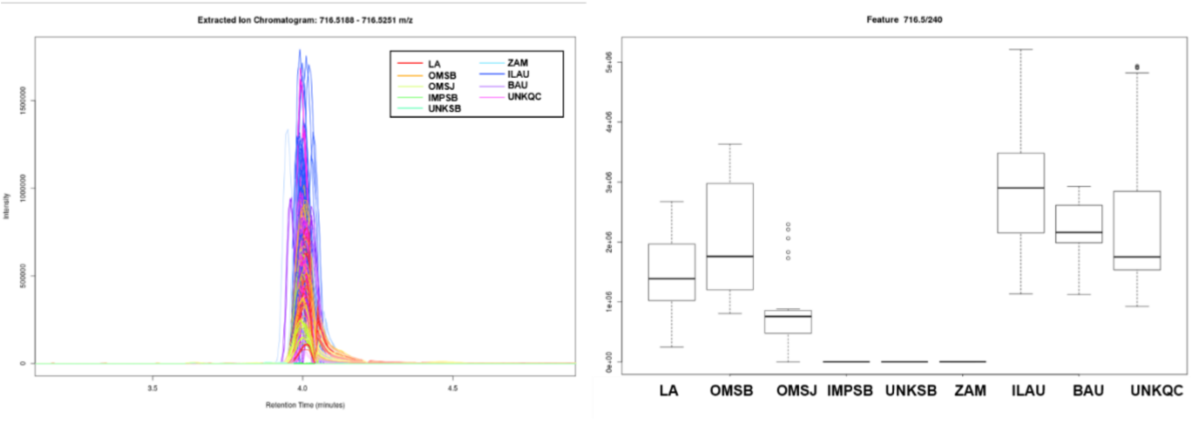

S9 Figure 9. Extracted ion chromatogram (left) for feature 716.5251 was identified by GNPS as a lipid derivative. The METLIN Database also matches this to 1-palmitoyl-2-linoleoyl phoshoethanolamine (3.0 ppm error), although GNPS marks this as a phosphocholine lipid. Box-and-whisker (right) representation shows that this compound is present in all local samples including UNKQC, but not in imported garlic. This compound could be an attribute feature of Philippine garlic.
