# Supplementary material for: Small in size, big on taste: Metabolomics analysis of flavor compounds from Philippine garlic
Source: PLoS One. 2021 May 20;16(5):e0247289. doi: 10.1371/journal.pone.0247289 (PMC8136657; doi:10.1371/journal.pone.0247289)
Supplement: S1 Table — (PDF) [file pone.0247289.s010.pdf]

S1 Table 1. Morphometric measurements of native and imported *Allium sativum* cultivars.

Sampling Location	Abbr.	Height (mm, %CV)	Width (mm, %CV)	Thickness (mm, %CV)	cloves per bulb
Batanes White	BAU	21.83, 8.78%	9.85, 15.22%	9.85, 13.45%	9-12
Ilocos White	ILAU	20.20, 10.55%	8.50, 12.67%	9.29, 12.81%	18-22
Laoag, Ilocos Norte	LA	20.67, 9.27%	8.27, 13.69%	9.33, 17.38%	19-23
Sablayan, Occidental Mindoro	OMSB	22.99, 14.14%	11.43, 17.56%	9.38, 12.38%	20-30
San Jose, Occidental Mindoro	OMSJ	22.67, 5.44%	9.30, 11.46%	9.87, 8.87%	20-24
Zamboanga City, Zamboanga Del Sur	ZAM	30.20, 9.85%	10.10, 12.08%	10.90, 13.12%	12-14

Sablayan, Occidental Mindoro	IMPSB	29.20, 9.27%	11.00, 15.92%	10.60, 9.89%	11-13
Quezon City, National Capital Region	UNKQ C	32.18, 7.33%	13.23, 11.22%	11.6, 9.75%	10-13
Unknown Sample, Sablayan, Occidental Mindoro	UNKSB	29.45, 5.85%	11.10, 10.57%	10.75, 10.80%	10-12
