# Supplementary material for: Small in size, big on taste: Metabolomics analysis of flavor compounds from Philippine garlic
Source: PLoS One. 2021 May 20;16(5):e0247289. doi: 10.1371/journal.pone.0247289 (PMC8136657; doi:10.1371/journal.pone.0247289)
Supplement: Attachment — Submitted filename: Response-PLOS-Edited.pdf [file pone.0247289.s011.pdf]

Secondary Metabolites Profiling Laboratory
2F Institute of Chemistry Research Wing
National Science Complex
University of the Philippines Diliman

30 April 2021

Dr. Timothy J. Garrett
Academic Editor
PLOS One

Dear Dr. Garrett,

Thank you very much for your review on our work, entitled "**Small in size, big on taste: metabolomics analysis of flavor compounds from Philippine garlic**" by K.F. Rellin, R.J.E. Molino, R. Nellas, and H. Junio. In this letter, we detail our response to the comments made by the reviewers.

From the Editor:

Comment: *Please ensure that your manuscript meets PLOS One's style requirements, including those for file naming.*

Response: The revised manuscript was edited accordingly.

Comment: *Please review your reference list to ensure that it is complete and correct. If you have cited papers that have been retracted, please include the rationale for doing so in the manuscript text or remove these references and replace them with relevant current references.*

Response: The reference list was checked and edited according to the journal's specifications.

Comment: *We note that S1 Figure 1 in your submission contains map images which may be copyrighted.*

Response: We have decided to remove the image to avoid complications with copyright.

Comment: *If applicable, we recommend that you deposit your laboratory protocols in protocols.io to enhance the reproducibility of your results. Protocols.io assigns your protocol its own identifier (DOI) so that it can be cited independently in the future.*

Response: We have decided not to deposit our protocols on this web-platform.

From Reviewer 1:

Comment: *I suggest the author should write the full name of the technique "Liquid chromatography-mass spectrometry" before the acronym LC-MS.*

Response: This portion of the manuscript was edited as suggested.

Comment: *Did the author use any sample normalization method for the analyses? Pre-normalization is important for uniform samples across the study. Did the author use, for example, sample mass for pre-normalization? This information needs to be explained at the method section.*

Response: Yes, an important normalization step was performed pre-acquisition in LC-MS. Details with regards to sample normalization method were clarified in the revised manuscript. Please refer to lines 146-147 on page 5 of the revised version.

Comment: *Did the author use Quality Control (QC) procedure or internal standards for monitoring the precision of the analytical process involving untargeted metabolomics? This information needs to be explained at the method section.*

Response: Yes. We have used quality control samples and internal standards for the analysis and have edited the method section to reflect this (lines 110-112, page 4; lines 135-136, page 5).

Comment: *The author observed the absence of the bioactive compound Allicin in the samples. Could this absence be due a possible inactivation of the allicin-producing enzyme, Alliinase, a naturally occurring enzyme present in the vascular bundle cells of the garlic bulb that is released when the bulb tissue is crushed?*

Response: We are not ruling out the possibility of enzymatic action that could inhibit the production of allicin. However, allicin is a volatile compound and its detection is atypical with LC-MS (lines 252-258, page 12).

Comment: *Identification of metabolites without authentic standards means that all metabolites have a very low confidence in identification. Thus, making any biological decisions from those metabolites is very difficult. It is recommended that you follow the metabolomics standards initiative for identification and that key metabolites are identified with standards that can match m/z value, retention time and MS/MS spectra if possible.*

Response: We acknowledge the limitations of our identification process, however, majority of the compounds we have putatively annotated were categorized as 'gold' in the GNPS reference library. These library spectra are derived from purified compounds with NMR and crystallographic data (lines 190-192, page 8).

Moreover, we have cross-referenced and verified our experimental spectra with those reported in literature that have previously isolated, identified, and characterized the compounds via MS and even NMR (lines 230-236, page 10-11).

From Reviewer 2:

Comment: *Not sure how the terms taste and flavor are being used here. Taste should refer to sensations on the tongue (sweet, salty, bitter, sour, umami), while flavor should refer to sensations via retronasal olfaction. Looking through the introduction and references, many of the chemical compounds referred to as influencing taste and flavor have little direct evidence of influencing taste and flavor in garlic through experiments using psychophysics of sensory science. Maybe this point was missed, but it seems many of the compounds are precursors to compounds shown to have taste and flavor functions, like Alliin is a volatile precursor.*

Response: We put great emphasis on gamma-glutamyl dipeptides. Their association with taste is well established through studies that looked at its interaction with nasal/oral targets such as Calcium sensing receptors (CaSRs). These results have also been correlated to sensory evaluation of gamma-glutamyl compounds and their taste-enhancing benefits (Yang, et. al 2019, Toelstede, et al. 2009, Dunket et al., 2007, Yang, et. al, 2021). Based on these studies, review articles describe gamma-glutamyl dipeptides as taste-enhancing compounds that are responsible for the elevated richness and mouthfulness taste of food (Yang, et. al, 2019). However, some studies (Ueda et al., 1990; Nakabayashi et al,. 2016; Amino et al., 2018) which provided data on CaSR assay and sensory evaluation on the compounds isolated from garlic, flavor-modifying was the preferred descriptor.

Comment: *It is unclear if 5 bio reps with 5 tech reps per bio rep was used. Please clearly state experimental setup.*

Response: The number of biological and technical replicates for each garlic cultivar were clarified in the revised manuscript (lines 99-100, page 3; lines 120-121, page 4).

Comment: *Why was a non-parametric analysis chosen?*

Response: We have added a clarification in the revised manuscript (lines 151-153, page 7). For untargeted metabolomics data, variances within the population may not be equal. Hence, as powerful as parametric analysis is, it would not be robust enough to handle the inherent heteroscedasticity of LC-MS data.

Comment: *How were the cultivars authenticated?*

Response: Based on the information provided by the Department of Agriculture Region II, cultivars are authenticated based on seed or clove morphology. The Batanes native cultivar has 12 or more smaller cloves, similar to the Ilocos native variety. Most imported cultivars that have been naturalized in the Philippines have bigger but fewer cloves per bulb (lines 96-98, page 3).

Comment: *I understand qTOF was used but why were no authentic standards used to ensure identification quality?*

Response: Same answer to the related question of Reviewer 1.

We are grateful of the positive and thorough assessment of our work. We thank you for your suggestions that helped us improve our manuscript.

We hope for your favorable response.

Sincerely,

Hiyas A. Junio, PhD
Associate Professor
Institute of Chemistry
University of the Philippines, Diliman
